# A single mutation in the *GSTe2* gene allows tracking of metabolically based insecticide resistance in a major malaria vector

**DOI:** 10.1186/gb-2014-15-2-r27

**Published:** 2014-02-25

**Authors:** Jacob M Riveron, Cristina Yunta, Sulaiman S Ibrahim, Rousseau Djouaka, Helen Irving, Benjamin D Menze, Hanafy M Ismail, Janet Hemingway, Hilary Ranson, Armando Albert, Charles S Wondji

**Affiliations:** 1 Vector Biology Department, Liverpool School of Tropical Medicine, Pembroke Place, Liverpool L3 5QA, United Kingdom; 2 Departamento de Cristalografía y Biología Estructural, Instituto de Química-Física “Rocasolano”, CSIC, C/Serrano 119 Madrid E-28006, Spain; 3 International Institute of Tropical Agriculture, 08 BP 0932 Cotonou, Benin; 4 Organisation de Coordination pour la lutte contre les Endémies en Afrique Centrale, PO Box 288, Yaoundé, Cameroon

## Abstract

**Background:**

Metabolic resistance to insecticides is the biggest threat to the continued effectiveness of malaria vector control. However, its underlying molecular basis, crucial for successful resistance management, remains poorly characterized.

**Results:**

Here, we demonstrate that the single amino acid change L119F in an upregulated glutathione S-transferase gene, *GSTe2*, confers high levels of metabolic resistance to DDT in the malaria vector *Anopheles funestus*. Genome-wide transcription analysis revealed that *GSTe2* was the most over-expressed detoxification gene in DDT and permethrin-resistant mosquitoes from Benin. Transgenic expression of *GSTe2* in *Drosophila melanogaster* demonstrated that over-transcription of this gene alone confers DDT resistance and cross-resistance to pyrethroids. Analysis of *GSTe2* polymorphism established that the point mutation is tightly associated with metabolic resistance to DDT and its geographical distribution strongly correlates with DDT resistance patterns across Africa. Functional characterization of recombinant *GSTe2* further supports the role of the L119F mutation, with the resistant allele being more efficient at metabolizing DDT than the susceptible one. Importantly, we also show that *GSTe2* directly metabolizes the pyrethroid permethrin. Structural analysis reveals that the mutation confers resistance by enlarging the GSTe2 DDT-binding cavity, leading to increased DDT access and metabolism. Furthermore, we show that *GSTe2* is under strong directional selection in resistant populations, and a restriction of gene flow is observed between African regions, enabling the prediction of the future spread of this resistance.

**Conclusions:**

This first DNA-based metabolic resistance marker in mosquitoes provides an essential tool to track the evolution of resistance and to design suitable resistance management strategies.

## Background

Insecticide-based control interventions, notably long lasting insecticide nets (LLINs) and indoor residual spraying (IRS), are key components of malaria control in Africa. Unfortunately, increasing resistance to available insecticide classes across Africa in major malaria vectors, such as the mosquito *Anopheles funestus*, is threatening the continued effectiveness of these control tools. Elucidating the molecular basis of insecticide resistance in these vectors is crucial to designing suitable resistance management strategies and preventing potentially devastating public health consequences [1].

Pyrethroids are the only class of insecticide used for LLIN impregnation, and are also the predominant insecticide class used in IRS, while dichlorodiphenyltrichloroethane (DDT) is still retained for use in IRS, due to the limited number of cost-effective alternatives. The two major causes of pyrethroid and DDT resistance are target-site insensitivity and metabolic resistance [2]. Target-site resistance (knockdown resistance (*kdr*)) can be easily monitored by PCR, while metabolic resistance is not easily tracked due to its complex molecular basis, despite its greater operational impact on malaria control [1]. The detailed molecular and structural basis through which candidate metabolic resistance genes confer resistance remains poorly characterized [3-5]. No single metabolic resistance marker has been identified in malaria vectors. Consequently, there is still no DNA-based diagnostic tool to detect metabolic resistance easily in field populations unlike target site resistance. Such tools are needed to detect and track resistance at an early stage allowing control programs to design rational, evidence-based resistance management strategies.

To address this gap in our knowledge, we dissected the molecular basis of metabolic resistance in a DDT/pyrethroid-resistant *An. funestus* population from Benin that is *kdr*-free [6]. Using genome-wide transcriptional and functional analyses combined with structural and population genetics studies, we conclusively demonstrated that a single amino acid change in the binding pocket of the glutathione-s-transferase epsilon 2 (*GSTe2*) gene, coupled with increased transcription, confers a high level of DDT resistance and also cross-resistance to pyrethroids in the major African malaria vector *An. funestus*.

## Results and discussion

### DDT resistance profile of *Anopheles funestus* in Benin and Cameroon mosquitoes

The Pahou population (Benin) had previously been described as highly DDT resistant [6] with no mortality 24 hr after 1 hr of exposure. The WHO bioassays conducted in Kpome (Benin) in this study indicated that this *An. funestus* population, which is located approximately 100 km from Pahou, was also resistant to DDT, with only 5% mortality 24 hr after 1 hr of exposure to 4% DDT for females and 21% mortality for males. The population from Gounougou in Cameroon was also resistant to DDT, but at a moderate level, with 52% mortality for females and 65% for males.

### Genome-wide transcription microarray analysis

A comparative genome-wide transcription analysis using the 44,000-probe (60-mer) custom microarray chip previously designed by Riveron *et al*. [5] was successfully used to identify the set of genes associated with DDT resistance in Pahou (Benin). A total of 1,352 probes were differentially expressed (≥ twofold at *P* < 0.01) between the DDT-resistant samples from Pahou and the susceptible strain FANG, with 321 upregulated and 1,031 downregulated in the Pahou samples compared with the susceptible strain FANG (Additional file 1: Figure S1A). The lists of the top probes that were differentially upregulated or downregulated are presented in Table 1 and Additional file 2: Table S1 respectively.

**Table 1:** **Upregulated genes in DDT-resistant Benin population of *Anopheles funestus *** **after microarray**

**Gene families**	**Gene name**	**Orthologs in *Anopheles gambiae***	**Corrected *P *** **value**	**Fold-change**	**Log**_**2 **_**fold-change**	**Description**
**Glutathione S-transferases**	combined_c920	AGAP009194	0.0076	11.9	3.6	Glutathione S-transferase gste2
	combined_c920	AGAP009194	0.0047	8.8	3.1	Glutathione S-transferase gste2
**Cytochrome P450s**	CYP6P9a	AGAP002865	0.0091	6.4	2.7	Cytochrome P450
	CYP6P9b	AGAP002865	0.0078	3.9	2.0	Cytochrome P450
**Carboxylesterases**	gb-COEAE1G	AGAP006700	0.0071	5.1	2.4	Alpha-esterase
**Short-chain dehydrogenases**	combined_c738	AGAP005166	0.0016	26.0	4.7	Short-chain dehydrogenase
	AGAP008125-RA	AGAP008125	0.0057	5.3	2.4	Short-chain dehydrogenase
	AGAP001405-RA	AGAP001405	0.0033	3.8	1.9	Short-chain dehydrogenase
	combined_c1061	AGAP011852	0.0079	3.7	1.9	Short-chain dehydrogenase
	AGAP001405-RA	AGAP001405	0.0035	3.7	1.9	Short-chain dehydrogenase
**Cuticle proteins**	AGAP005999-RA	AGAP005999	0.0048	10.8	3.4	Pupal cuticle
	AGAP006867-RA	AGAP006867	0.0034	5.2	2.4	Adult-specific cuticular protein acp-20
	AGAP010906-RA	AGAP010906	0.0038	3.6	1.9	Cuticle protein
	combined_c1722	AGAP010122	0.0061	5.0	2.3	Pupal cuticle protein
	combined_c1722	AGAP010122	0.0031	4.8	2.3	Pupal cuticle protein
	AGAP006867-RA	AGAP006867	0.0037	2.9	1.5	Adult-specific cuticular protein acp-20
**Odorant binding proteins**	AGAP001012-RA	AGAP001012	0.0038	8.2	3.0	Candidate odorant receptor
	combined_c1773	AGAP006368	0.0064	2.7	1.5	Odorant binding protein 4
	combined_c1773	AGAP006368	0.0072	2.7	1.4	Odorant binding protein 4
	AGAP009390-RA	AGAP009390	0.0082	2.1	1.1	Olfactory receptor
**Proteases**	AGAP004857-RA	AGAP004857	0.0036	5.2	2.4	Clip-domain serine proteinase
	gb-SP8905	AGAP003640	0.0057	4.0	2.0	Prolylcarboxypeptidase
	AGAP006674-RA	AGAP006674	0.0059	3.4	1.8	Serine protease
	combined_c1563	AGAP003581	0.0039	2.3	1.2	Chymotrypsin-like protein
	AGAP004904-RA	AGAP004904	0.0056	4.5	2.2	Catalase
	CD578028.1	AGAP006485	0.0081	3.8	1.9	Serine threonine-protein kinase rio1
**Transporters**	combined_c1762	AGAP006364	0.0094	2.8	1.5	ABC transporter
	combined_c3512	AGAP006186	0.0039	2.2	1.1	Calcium-transporting ATPase
	AGAP012626-RA	AGAP012626	0.0047	6.9	2.8	Serotonin transporter
**Heat shock proteins**	combined_c4173	AGAP001424	0.0021	11.6	3.5	Glycoprotein 93
	combined_c4173	AGAP001424	0.0049	9.6	3.3	Glycoprotein 93
	AGAP002076-RA	AGAP002076	0.0033	2.7	1.4	Heat shock protein cognate isoform a
**Salivary proteins**	EE589639.1	AGAP008281	0.0063	3.0	1.6	d7-related 1 protein
	EE590018.1	AGAP008216	0.0022	2.2	1.1	gsg7 salivary protein
	EE589541.1	AGAP008283	0.0086	2.1	1.1	d7-related 3 protein
**UDP (uridine diphosphate) glucosyltransferases**	combined_c1211	AGAP007920	0.0030	2.1	1.1	Glucosylglucuronosyltransferases
**Other functions**	combined_c1691	AGAP013141	0.0041	34.4	5.1	Mediator complex
	AGAP003209-RA	AGAP003209	0.0083	5.0	2.3	Sterol desaturase
	combined_c3636	AGAP001423	0.0031	3.7	1.9	Bifunctional purine biosynthesis protein
	combined_c3636	AGAP001423	0.0040	3.6	1.8	Bifunctional purine biosynthesis protein
	combined_c8340	AGAP003581	0.0047	2.7	1.4	Sorbitol dehydrogenase
	combined_c1577	AGAP000523	0.0047	2.1	1.1	ATP synthase lipid-binding mitochondrial precursor

The most upregulated detoxification gene in Benin was a glutathione S-transferase gene, *GSTe2*, with a fold-change (FC) of 11.9 (*P* = 0.0076) (Table 1 and Additional file 3: Table S2; Additional file 1: Figure S1A). The consistency of this upregulation is further supported by the fact that both probes designed for *GSTe2* were upregulated in the Pahou population (Table 1). Furthermore, orthologs of *GSTe2* were also upregulated in DDT-resistant strains in other mosquito species such as *An. gambiae*[3,7] and *Aedes aegypti*[8], suggesting that this gene most likely plays a key role in DDT resistance in many mosquito species.

The two duplicated cytochrome P450 genes, *CYP6P9a* (FC = 6.4) and *CYP6P9b* (FC = 3.9), which confer pyrethroid resistance in southern African populations [5], were also upregulated in the Benin population. However, because their encoded proteins are unable to metabolize DDT [5], they are most likely associated with the permethrin resistance observed in the Pahou mosquitoes. Other genes with a known association with insecticide resistance were also upregulated in Pahou mosquitoes and are detailed in Additional file 4 Supplementary text and listed in Table 1.

### Validation of the microarray upregulation with quantitative RT-PCR

**Figure 1:**
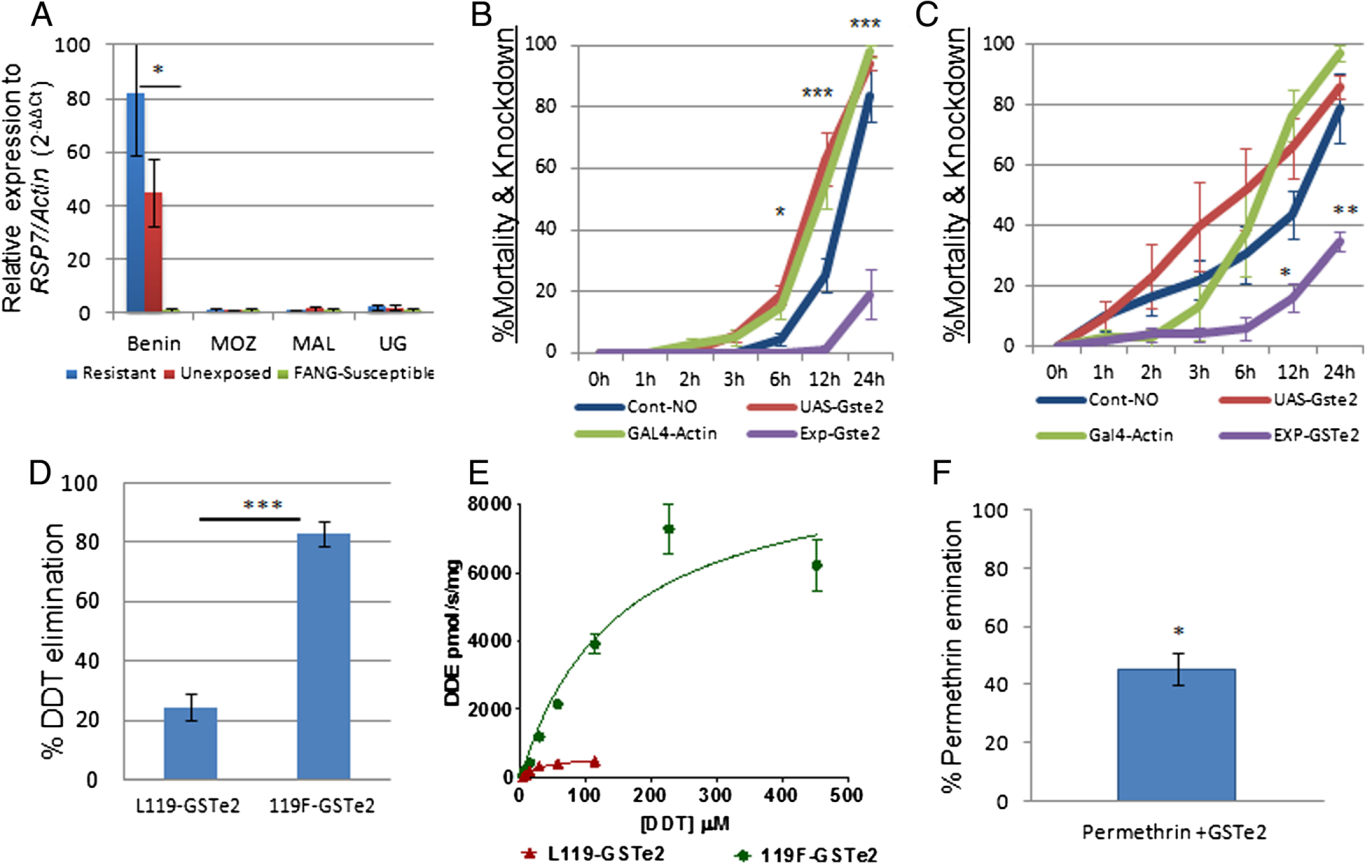
***GSTe2 *expression and functional analysis. (A)** Comparative qRT-PCR examining DDT-resistant (Benin) and DDT-susceptible (Mozambique, Malawi and Uganda) mosquitoes. **(B)** DDT bioassay tests on transgenic Act5C-GSTe2 flies (Exp-GSTe2) and control strains (two parental (UAS-GSTe2 and GAL4-actin) and F_1_ progeny that do not express the GSTe2 transgene (Cont-NO)). **(C)** The same bioassays with permethrin. **(D)** DDT metabolic activity (depletion rate after 1 hr) of GSTe2 alleles (mean ± standard deviation). **(E)** Michaelis–Menten enzyme kinetics for resistant and susceptible GSTe2 alleles **(F)** Permethrin metabolic activity (depletion rate after 1 hr) for the 119 F GSTe2 allele (mean ± standard deviation). DDT, dichlorodiphenyltrichloroethane; MAL, Malawi; MOZ, Mozambique; qRT-PCR, quantitative reverse transcriptase polymerase chain reaction; UG, Uganda, DDE, dichlorodiphenyldichloroethylene.

Quantitative reverse-transcriptase PCR (qRT-PCR) confirmed the significant upregulation of *GSTe2* in Benin (FC = 44.8, *P* = 0.007) (Additional file 1: Figure S1B) in comparison to the FANG susceptible strain. The *GSTe2* gene is significantly over-transcribed in DDT-exposed mosquitoes compared to unexposed mosquitoes (FC = 82.0 vs 44.8, *P* < 0.05) (Figure 1A), suggesting that induction of *GSTe2* occurs in addition to constitutive over-expression in resistant mosquitoes. The expression pattern of *GSTe2* across Africa significantly correlates with DDT-resistance patterns. This gene is 69.5- and 79.1-fold upregulated in highly DDT-resistant mosquitoes from Benin (*P* < 0.05) compared with the fully DDT-susceptible Mozambique and Malawi samples, respectively (Figure 1A). It is 35-fold upregulated (*P* < 0.05) compared to Ugandan samples (moderate DDT resistance [9]). Similarly, the two P450s *CYP6P9a* and *CYP6P9b* had 2.96 and 7.13 times more expression in mosquitoes from Pahou than in the susceptible FANG mosquitoes.

Overall, transcription analyses indicated that *GSTe2* is the main detoxification gene associated with DDT resistance in Pahou mosquitoes. In other mosquito species, DDT resistance is conferred by additional mechanisms, such as the knockdown resistance (*kdr*) mutation (*An. gambiae*[10] and *Aedes aegypti*[8]), or by upregulation of cytochrome P450s, as observed for *An. gambiae* for *CYP6Z1*[11] and *CYP6M2*[12]. None of these mechanisms are present in *An. funestus* because no *kdr* mutation was detected in this population [6], and the only upregulated P450s in this population (*CYP6P9a* and *CYP6P9b*) are unable to metabolize DDT [5]. These results indicate that *GSTe2* is the main detoxification gene associated with DDT resistance in this Benin population of *An. funestus*. Therefore, further analysis of this gene would be very good for elucidating metabolic resistance mechanisms and for understanding the detailed molecular basis through which it confers resistance to insecticides.

### Transgenic expression of *GSTe2* in *Drosophila* flies

To establish whether the upregulation of *GSTe2* alone can confer DDT and pyrethroid resistance, transgenic *Drosophila melanogaster* flies expressing *GSTe2* cloned from Benin were generated using the GAL4/UAS system under the ubiquitous Act5C-GAL4 driver (Act5C-GSTe2). After confirming the expression of *GSTe2* in transgenic F_1_ progeny by qRT-PCR (Additional file 1: Figure S1C), bioassays revealed that the transgenic Act5C-GSTe2 flies were resistant to 4% DDT (19.1% mortality after 24 hr exposure), whereas, all the controls that did not over-express *GSTe2* were susceptible (83.5% to 97.9% mortality, *P* < 0.0001) (Figure 1B). These results indicate that *GSTe2* upregulation alone is sufficient to confer DDT resistance. Such a transgenic expression approach was also recently successfully used to confirm the involvement of two P450s (*CYP6P9a* and *CYP6P9b*) in conferring pyrethroid resistance in *An. funestus*[5] and previously in *Drosophila*[13], indicating the usefulness of this technique.

Noticeably, the upregulation of *GSTe2* also conferred cross-resistance to pyrethroids. Indeed, bioassays performed with 2% permethrin revealed that the transgenic Act5C-GSTe2 flies (35.6% mortality after 24 hr exposure) but not the controls (78.9% to 97.2% mortality, *P* < 0.001) (Figure 1C) were permethrin resistant. A moderately reduced mortality rate (*P* < 0.05) was observed in transgenic flies after 0.15% deltamethrin exposure only after 24 hr (Additional file 1: Figure S1D). However, the mortality rate obtained for pyrethroids was higher than for DDT, suggesting that the resistance conferredto pyrethroids by *GSTe2* is lower than to DDT. Such observations are in accordance with the resistance profile of the Pahou population, which is highly resistant to DDT but only moderately resistant to permethrin [6]. This potential role of *GSTe2* in permethrin resistance is in agreement with previous reports that suggested that orthologs of *GSTe2* in other mosquitoes are associated with pyrethroid resistance. Indeed, microarray analyses of *An. gambiae*[3] reported upregulation of *GSTe2* in a permethrin-resistant strain, with the suggestion that the protein for this gene could be involved in permethrin resistance either by acting as a pyrethroid-binding protein and sequestering the insecticide [14] or by protecting mosquitoes against the oxidative stress and lipid peroxidation caused by exposure to permethrin [15]. In the dengue fever mosquito *Ae. aegypti*, a partial knockdown of the ortholog of *GSTe2* led to increasing mortality to permethrin, indicating that *GSTe2* is also associated with permethrin resistance in this species [8]. This cross-resistance to pyrethroids is of significant concern for malaria control as *GSTe2* could protect mosquitoes against the major insecticides used in public health.

### Detection of resistance markers in *GSTe2*

**Figure 2:**
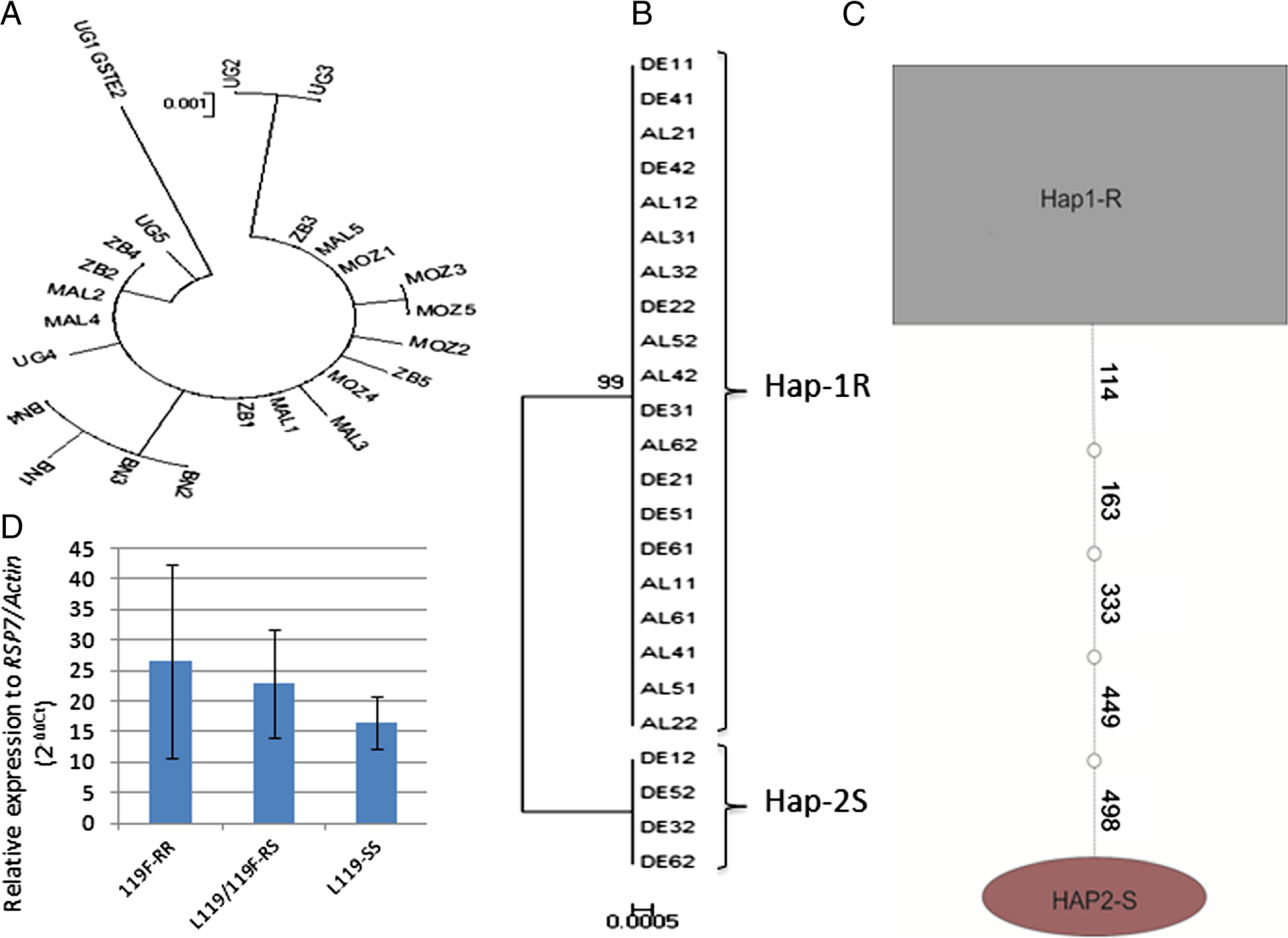
***GSTe2 *polymorphism and DDT resistance. (A)** Maximum likelihood tree of *GSTe2* cDNA across Africa. **(B)** Same as in (A), but with DDT-resistant (AL) and DDT-susceptible (DE) mosquitoes in Benin (genomic DNA). **(C)***GSTe2* haplotype network (TCS) between susceptible and resistant mosquitoes (Benin). The polygon size reflects the haplotype frequency. Each node represents a mutation (number). **(D)***GSTe2* expression profile of the three L119F genotypes in Cameroon (Gounougou). BN, Benin; DDT, dichlorodiphenyltrichloroethane; MAL, Malawi; UG, Uganda; ZB; Zambia.

To detect resistance markers for *GSTe2*, we analyzed the full-length cDNA (666 bp) from resistant Benin mosquitoes, and four susceptible mosquito strains from Uganda, Malawi, Mozambique and Zambia. There were 19 polymorphic sites (8 replacement substitutions) from 24 clones. The major difference in the Benin strain was a fixed leucine (CTT) to phenylalanine (TTT) replacement (L119F). The Benin haplotypes form a unique clade compared with the haplotypes from the other countries (Figure 2A).

Analysis of the full-length *GSTe2* (881 bp including introns) of six resistant and six susceptible mosquitoes from another Benin location (Kpome, 6°55′N, 2°19′E, because no susceptible mosquitoes were available from Pahou) confirmed that the *GSTe2* polymorphism was associated with DDT resistance. No polymorphisms were identified for the resistant mosquitoes and there was a single haplotype (Hap1-R) bearing the resistant 119 F allele (Figure 2B,C; Additional file 3: Table S2). Five heterozygote polymorphic sites were identified for susceptible mosquitoes with a single amino acid change, L119F. The presence of a susceptible haplotype Hap2-S bearing the L119 susceptible allele was also observed (Figure 2B,C).

### Correlation between the L119F mutation and DDT and pyrethroid resistance

**Figure 3:**
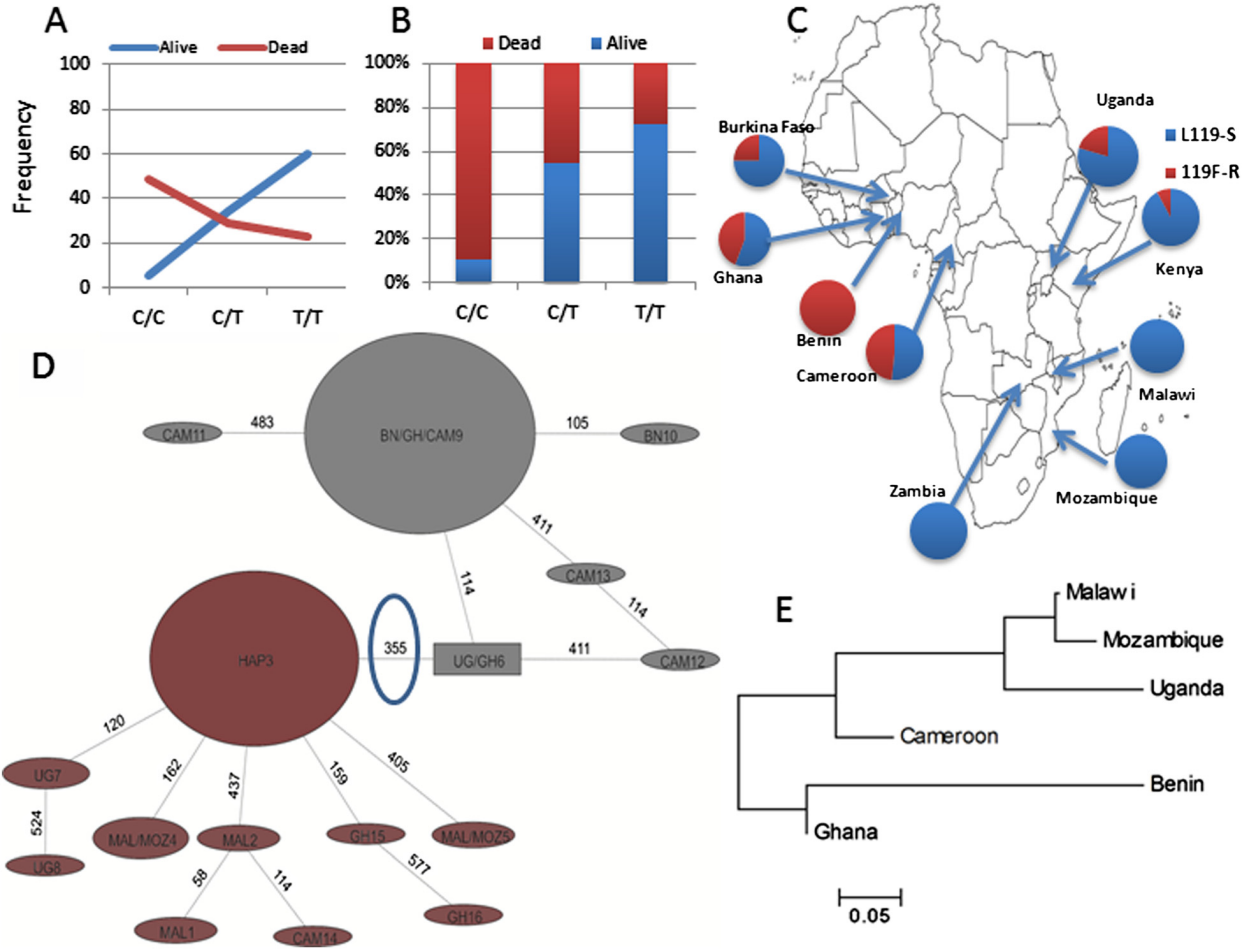
**L119F association with DDT resistance and *GSTe2 *variability across Africa. (A)** Evidence of the strong correlation between L119F genotypes and DDT resistance in Gounougou (Cameroon) (25 resistant and 25 susceptible). **(B)** Differential distribution of L119F genotypes between DDT-resistant and DDT-susceptible mosquitoes. **(C)** The geographical distribution of L119F across Africa correlates with the DDT resistance distribution. **(D)***GSTe2* haplotype network (TCS) (coding regions only) across Africa, with resistant (grey) and susceptible (maroon) clades defined by L119F. Each haplotype is represented by a circular or rectangular shape and the size proportional to its frequency in the sample while mutational steps are represented by line numbers. **(E)** Genetic distances between six African populations based on *GSTe2* sequences (*Nst* estimates). DDT, dichlorodiphenyltrichloroethane.

The TaqMan assay, designed to genotype the L119F mutation, unambiguously detected the three genotypes (Additional file 5: Figure S2A) in 35 susceptible and 35 resistant mosquitoes from Gounougou (9°05′N, 13°40′E) in Cameroon (west-central Africa) (as very few susceptible mosquitoes were obtained from Benin). The distribution of the genotype frequencies between susceptible and resistant mosquitoes significantly differed (χ^2^ = 99.7, degrees of freedom = 2 and *P* < 0.0001) (Figure 3B). A significant association was observed between the L119F mutation and DDT resistance, with an odds ratio of 5.74 (*P* < 0.0001) when comparing the frequency of the resistant and susceptible alleles in both sample sets. When comparing the frequency of the two homozygous genotypes (T/T and C/C), the T/T mutant genotype was more highly associated with DDT resistance than the C/C wild genotype with an odds ratio of 22.3 (*P* < 0.0001). Indeed, 72.5% of the mosquitoes with the homozygote mutant allele (T/T) were resistant to DDT whereas approximately half of the heterozygote mosquitoes were resistant to DDT (54.5%), and only 10% of the homozygote wild-type (C/C) were resistant to DDT, suggesting co-dominance of this allele (Figure 3B). The correlation of the L119F mutation with DDT resistance indicates that this TaqMan assay could be used as a diagnostic tool to detect and map the distribution of DDT resistance in *An. funestus* in Africa.

This is the first report to our knowledge of a point mutation conferring metabolic resistance in a mosquito species. To date, metabolic resistance to DDT has only been associated with a single haplotype of the P450 gene *CYP6G1* in *D. melanogaster*[16] or to a potential allelic variation in the ortholog of *GSTe2* in *Ae. aegypti*[8] but not to a single point mutation as detected in this study in *An. funestus*. Such a point mutation conferring metabolic resistance has previously only been described for the house fly (*Musca domestica*) for the alpha esterase E7 gene [17] and for the sheep blowfly (*Lucilia cuprina*) for the carboxylesterase E3 gene [18]. Detection of the L119F mutation in *An. funestus*, which confers high DDT resistance, indicates that similar point mutations conferring resistance to other insecticide classes could also be found in mosquitoes, notably in malaria vectors, allowing the design of reliable molecular diagnostic assays that can accurately detect and track metabolic resistance in the field. Although a similar trend was observed for the pyrethroids permethrin and lambda-cyhalothrin, the correlation between L119F and resistance to these insecticides was not significant (*P* = 0.08 for permethrin, *P* = 0.1 for lambda-cyhalothrin) (Additional file 5: Figure S2C,D). Hence, the cross-resistance to pyrethroids may be primarily conferred by the quantitative change in *GSTe2* expression rather than the qualitative change from the L119F mutation.

In contrast, DDT resistance was more closely associated with the L119F mutation than the level of *GSTe2* over-expression (Figure 2D). Indeed, despite a lower fold-change in the susceptible L119 genotype, no significant difference (*P* > 0.05) in the *GSTe2* expression level was observed between the homozygote resistant RR (FC = 26.6), the heterozygote RS (FC = 23) and the homozygote susceptible SS (FC = 16.5) (Figure 2D). These results suggest that the L119F mutation could be the predominant cause of DDT resistance.

### Geographical distribution of L119F across Africa

Genotyping 30 mosquitoes from each of nine African countries revealed that the geographical distribution of the 119 F resistant allele strongly correlates with the distribution of DDT resistance across Africa [19,20] (Figure 3C; Additional file 5: Figure S2B). The 119 F resistant allele is fixed in highly DDT-resistant Benin mosquitoes but completely absent in fully susceptible southern African populations [5,21,22]. It also occurs in other DDT-resistant populations in West Africa with a frequency of 48.2%, 44.2% and 25%, respectively, in Cameroon, Ghana and Burkina Faso, in correlation with the previously reported prevalence of DDT resistance in these countries [19,20]. The resistant 119 F allele has also been detected in the eastern African countries of Uganda (20.4%) and Kenya (7.8%) but with lower frequencies, reflecting the moderate level of DDT resistance that was previously reported [9]. The L119F distribution in Africa is similar to that of the A296S resistance-to-dieldrin (RDL) mutation, which confers dieldrin resistance [20] and probably reflects contemporary patterns of gene flow between *An. funestus* populations across Africa. Indeed, as for L119F, no resistance allele was found for RDL in southern Africa in line with the full susceptibility to dieldrin in this region. Similarly, as for L119F, only a low frequency of RDL was detected in Uganda in east Africa.

### Metabolic assays with heterologous GSTe2 enzyme

An assessment of the DDT dehydrochlorinase activity of the recombinant 119 F resistant enzyme (Benin) and that of susceptible L119 revealed that the 119 F allele is 3.4 times more efficient at metabolizing DDT (82.9% DDT depletion after 1 hr reaction in the presence of the cofactor glutathione (GSH); *P* < 0.001) than the L119 allele (24.4% depletion) (Figure 1D and Additional file 6: Figure S3A). This is confirmed by their respective kinetic parameters (Figure 1E), with the 119 F allele having a higher catalytic efficiency (kcat/*K*_*m*_ ratio) for DDT (316.3 μM^−1^.s^−1^ vs 92.0 μM^−1^.s^−1^) (Table 2).

**Table 2:** **Kinetic parameters of the resistant (119 F) and susceptible (L119) *GSTe2 *** **alleles**

	***V***_**max **_**(DDE μmol.min**^**−1**^**.mg**^**−1**^**)**	***K***_***m ***_**(μM DDT)**	**Kcat (s**^**−1**^**)**	**Kcat/*K***_***m ***_**(μM**^**−1**^**.s**^**−1**^**)**
119 F-GSTe2	60.8 ± 7.2	149.6 ± 39.6	47312 ± 5297	316.3
L119-GSTe2	3.75 ± 0.26	34 ± 5.9	3129 ± 222.6	92.0

Three independent assays were performed for both alleles and the results show mean ± standard deviation.

Additionally, significant permethrin metabolism was observed for the Benin 119 F-GSTe2 allele (45% depletion, *P* = 0.016) (Figure 1F; Additional file 6: Figure S3B and S3C) with the high-performance liquid chromatography (HPLC) metabolism profile showing three potential metabolite peaks. This suggests that *GSTe2* confers permethrin resistance by directly metabolizing this insecticide. Similarly, recent research has shown that a glutathione S-transferase (GST) from *Culex pipiens* (*CpGSTD1*) was able to metabolize pyrethroid-like fluorescent substrates directly, including a permethrin-like substrate [23]. The nature of the permethrin metabolites corresponding to the three peaks observed in this study remains unknown. Three potential metabolic pathways have been proposed for GST metabolism of pyrethroid-like substrates including halogen substitution, Michael addition, and thiolysis [23]. Because none of the three metabolite peaks in this study matched the permethrin ester hydrolysis products, phenoxybenzyl alcohol or phenoxybenzoic acid (produced by the Michael addition and the thiolysis routes), it is likely that a GSH conjugate to permethrin through the halogen substitution metabolic route could be the main mechanism. However, further investigations are needed to determine the nature of these metabolites. But it is possible that *GSTe2* could also confer permethrin resistance through other mechanisms such as sequestration [14] or protection against oxidative stress and lipid peroxidation [15].

In contrast, no significant metabolism was observed for deltamethrin, which is in line with the low deltamethrin resistance observed in the field in Pahou (88% mortality for deltamethrin vs 66% for permethrin), but also the lower deltamethrin resistance observed in transgenic UAS-GSTe2 *Drosophila* flies.

### Genetic diversity of *GSTe2* across Africa

#### Signature of positive selection on *GSTe2* in Benin

Analysis of the 882-bp full-length sequence of *GSTe2* (663 bp for the three exons and 219 bp for the two introns and part of the 3′ UTR) from field-collected mosquitoes from six countries revealed that *GSTe2* is under strong directional selection in Benin in contrast to other countries (Table 3 and Additional file 7: Table S3, Additional file 8: Figure S4A). Indeed, the significant reduction of genetic diversity of *GSTe2* in Benin is apparent with only one polymorphic site observed in a single mosquito out of 12 from Pahou, in contrast to the seven to nineteen polymorphic sites in the other populations. Only two haplotypes were present in Pahou mosquitoes (the minor haplotype was only present as a heterozygote in one out of twelve individuals), in contrast to the seven to ten haplotypes for the other countries (Additional file 8: Figure S4A; Table 3). Genetic diversity parameters such as haplotype diversity (hd) and nucleotide diversity for Benin are significantly lower than for the other five countries (Additional file 8: Figure S4A). This reduced diversity of *GSTe2* for Benin mosquitoes suggests the presence of a selective sweep across and around this gene. A future assessment will establish the extent of this selective sweep in this genomic region on chromosome 2 L.

**Table 3:** Genetic parameters between coding and non-coding regions for all three genes in the three countries

		**Complete sequenced fragment (882 bp)**							**Coding region (663 bp)**							**Non-coding region (219 bp)**				
**Gene**	**2n**	**S**	**h**	**hd**	**π**	**θ**	**D**	D*	**S**	**h**	**hd**	**π**_**c**_	**θ**	**D**	D*	**S**	**h**	**π**_**nc**_	**D**	D*
**Benin**	24	1	2	0.08	0.09	0.3	−1.15 ns	−1.6 ns	1	2	0.08	0. 13	0.4	−1.15 ns	−1.6 ns	0	1	0	–	–
**Cameroon**	14	7	8	0.89	2.9	2.5	0.6 ns	0.21 ns	5	6	0.77	2.4	2.4	0.12 ns	−0.22 ns	2	3	4.2	1.3 ns	0.93 ns
**Ghana**	16	19	10	0.86	5.4	6.8	−0.8 ns	−0.38 ns	4	5	0.67	2.0	1.8	0.35 ns	0.25 ns	15	9	16	−1.1 ns	−0.55 ns
**Mozambique**	10	12	8	0.95	3.6	5.2	−1.4 ns	−1.4 ns	2	3	0.37	0.6	1.1	−1.4 ns	−1.6 ns	10	8	13	−1.3 ns	−1.25 ns
**Malawi**	18	14	7	0.78	4.4	4.6	−0.14 ns	1.14 ns	4	5	0.71	1.6	1.7	−0.28 ns	0.21 ns	10	6	13	−0.05 ns	1.41^*^
**Uganda**	10	17	9	0.97	7.5	6.8	0.5 ns	0.15 ns	3	4	0.71	1.7	1.6	−0.76 ns	−1.12 ns	14	7	26	0.72 ns	0.41 ns
**Total**	92	37	39	0.86	5.1	8.7	−1.3 ns	−0.33 ns	13	16	0.73	2.3	3.8	−1.07 ns	−0.8 ns	24	29	0.013	−1.3 ns	−0.06 ns

2n, number of sequences; D, Tajima’s statistics; D*, Fu and Li’s statistics; h, number of haplotypes; hd, haplotype diversity; ns, not significant; π, nucleotide diversity multiplied by 10^3^; S, number of polymorphic sites; θ, Watterson’s estimator (per site) multiplied by 10^3^.

However, other tests of selection such as the McDonald and Kreitman (MK), Hudson–Kreitman–Aguade (HKA), *dN*/*dS* and *K*_*a*_/*K*_*s*_ ratios and Tajima’s D tests did not show any signature for positive selection in Benin (Additional file 7: Table S3). This could be due to the fact that the sweep around *GSTe2* in the Benin population is nearing fixation as shown by the 100% frequency of the resistant 119 F allele in this population. In a situation of near fixation of the selective sweep, directional selection is better shown by reduced levels of genetic variation [24]. Evidence of selection through reduced genetic diversity was not observed in Cameroon and Ghana, where DDT resistance has also been detected, although not at the high level as in Benin. However, the presence of the predominant Benin-resistant haplotype in these west African countries, although at a lower frequency, could result from the combined effect of local DDT selection and migration.

Directional selection has previously been observed in this species in southern African pyrethroid-resistant populations at the two tandemly duplicated P450 genes *CYP6P9a* and *CYP6P9b*[5]. Another P450, *CYP6G1*, which confers DDT resistance in *D. melanogaster*, is also under strong directional selection, with a single resistant haplotype containing an Accord transposable element in the 5′ UTR region distributed globally [16]. The same *CYP6G1* gene is also under directional selection in *D. simulans*, with a 100-kb region having extensive reduced heterozygosity due to a selective sweep around *CYP6G1*[25]. These observations further suggest that insecticide resistance is an excellent example of natural selection at work. As expected, the non-coding regions exhibit greater genetic diversity than the coding regions in all countries, with more polymorphic sites and haplotypes apart from the Benin population, where the unique polymorphic site is in the coding region (Table 3).

#### Haplotype distribution of GSTe2 across Africa

A total of 39 haplotypes were observed for the full gene (Additional file 8: Figure S4B), 16 for coding regions only (Additional file 8: Figure S4C), and there were seven protein variants (Additional file 8: Figure S4D) across Africa. Resistant haplotypes (with the 119 F resistant allele) resolving around a predominant haplotype BN23 (nearly fixed in Benin and only present in DDT-resistant locations) all had reduced diversity, which is indicative of a recent selection with fewer haplotypes (11 out of 39), and more homogeneity (less mutational step differences ≤4). The susceptible haplotypes (with the L119 allele) resolved around a predominant haplotype MAL3 and were more diverse, with more haplotypes (29 out of 39) and greater heterogeneity (up to 13 mutational step differences). Overall, the haplotype distribution of *GSTe2* across Africa (Additional file 8: Figure S4B,C,D) reveals that the L119F mutation is the main polymorphism shaping *GSTe2* genetic diversity with the mutational step at this allele consistently defining a resistant and a susceptible group of haplotypes (Figure 3D and Additional file 9: Figure S5).

#### Population substructure of GSTe2 across Africa

**Figure 4:**
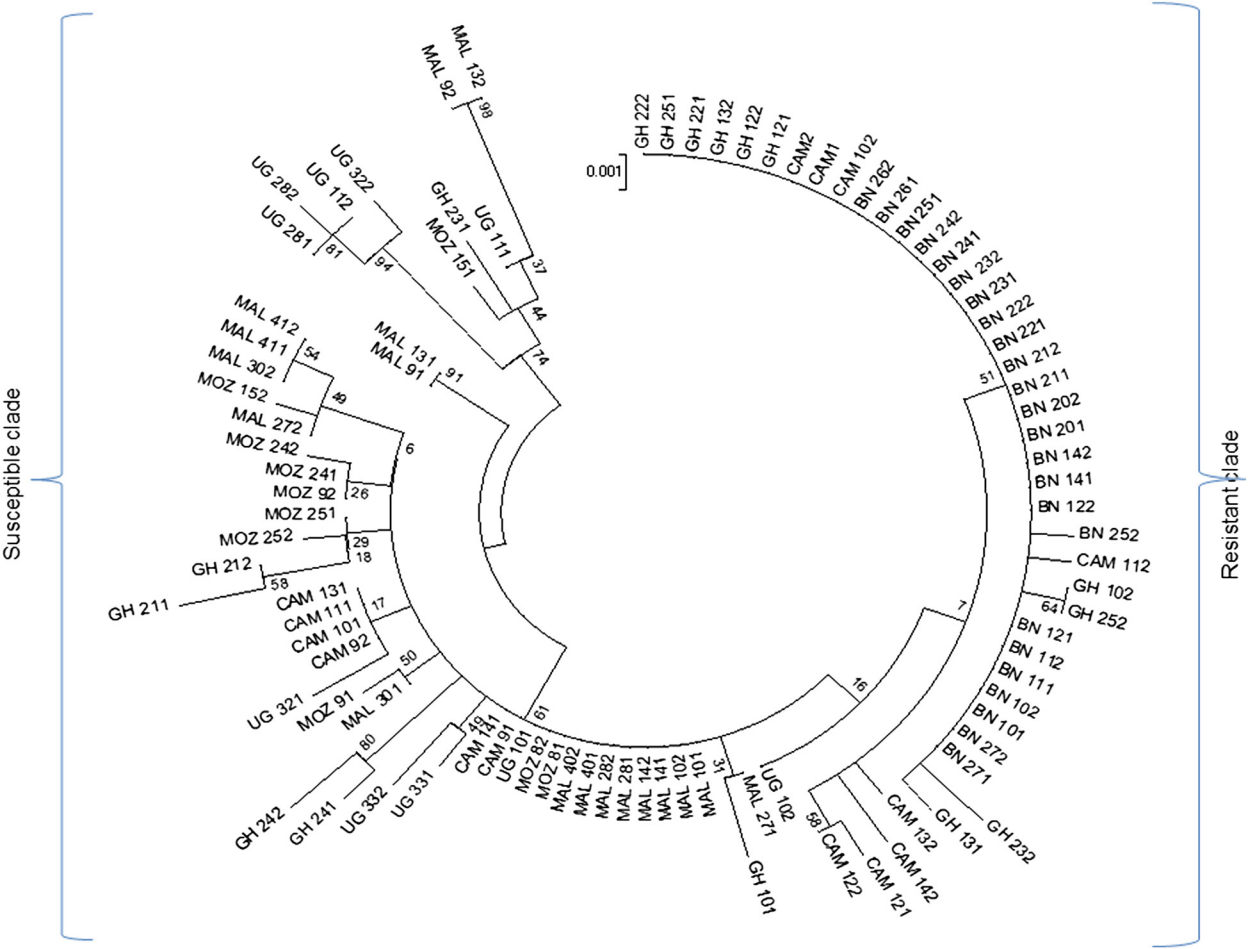
**Maximum likelihood phylogenetic tree of *GSTe2 *(coding and non-coding regions) across Africa.** The analysis involved 92 sequences (2n) labeled. All positions with gaps and missing data were eliminated. There were a total of 881 positions in the final dataset. A resistant clade that is less polymorphic and dominated by the Benin population is clearly identifiable to the right of the tree whereas a susceptible clade that is more polymorphic and more cosmopolitan is at the left of the tree. BN, Benin; CAM, Cameroon; GH, Ghana; MAL, Malawi; MOZ, Mozambique; UG, Uganda.

Analysis of the genetic diversity of *GSTe2* indicated that *An. funestus* populations are clearly structured according to their pattern of DDT resistance and by geographical distance. The construction of a maximum likelihood phylogenetic tree of *GSTe2* sequences revealed that Cameroon and Ghana mosquitoes cluster with Benin mosquitoes (Figure 4), correlating with DDT-resistance profiles. Susceptible populations from Mozambique and Malawi have the lowest genetic differentiation (*K*_*st*_ = 0.016) (Additional file 10: Table S4; Figure 3E). The moderately resistant east Africa Ugandan population has an intermediate differentiation to all populations. This pattern of gene flow correlates with the genetic structure patterns of *An. funestus* populations across Africa based on microsatellite markers [26], suggesting that there are barriers to gene flow between African *An. funestus* populations, which will affect the spread of insecticide-resistance genes.

### Structural basis of DDT resistance conferred by *GSTe2*

**Figure 5:**
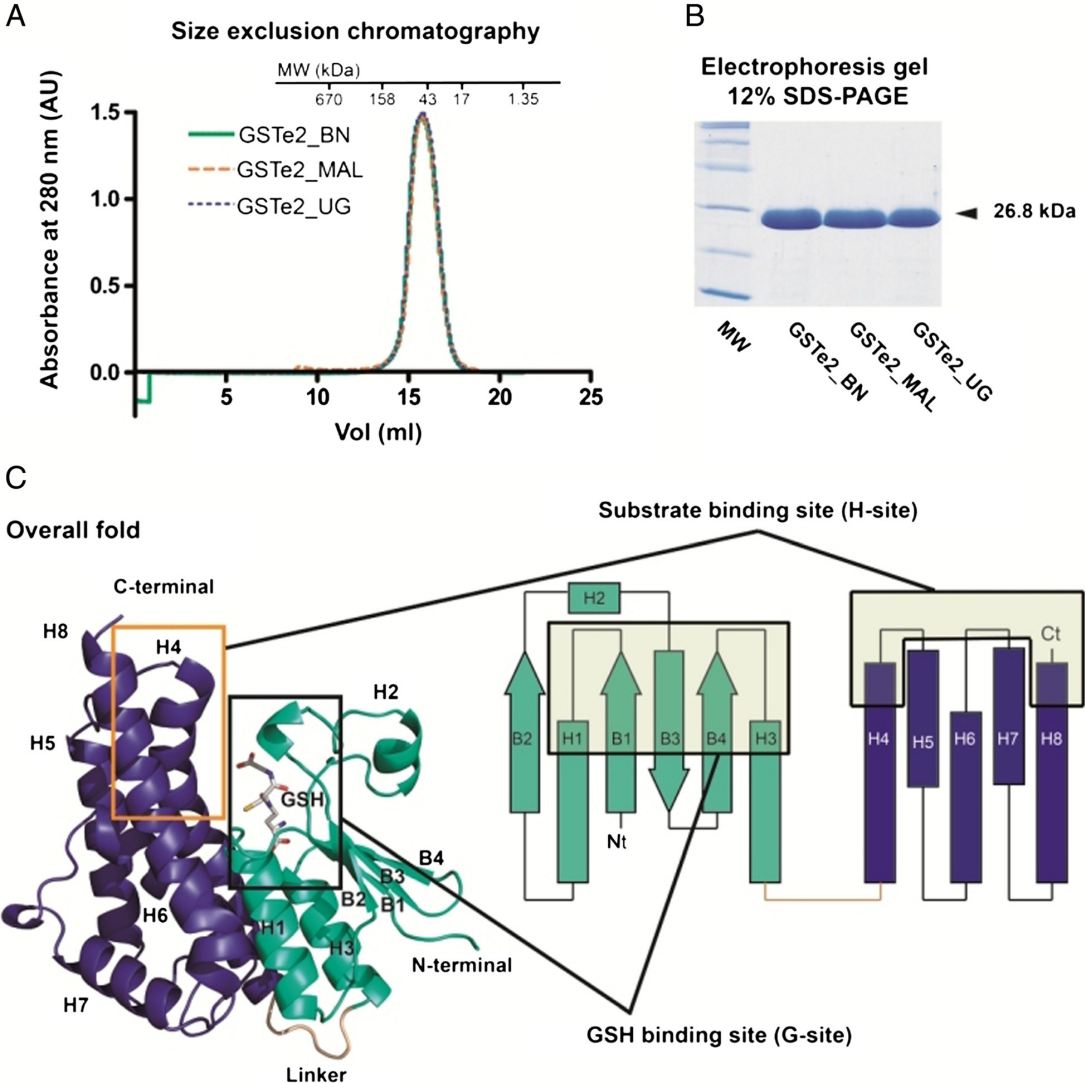
**GSTe2 purification and X-ray three-dimensional structure. (A)** Size-exclusion chromatogram of GSTe2 alleles from Benin (BN), Uganda (UG) and Malawi (MAL) alleles. The lines show the absorbance recorded at 280 nm. Molecular-weight markers (Bio-Rad, Hercules, CA, US) are indicated in kilodaltons. **(B)** SDS-PAGE gel of the three purified GSTe2 alleles (26.8 kDa). **(C)** Topology of GSTe2 showing the C- and N-terminals, the GSH binding pocket (G-site) and the substrate-binding pocket (H-site). AU, arbitrary units; BN, Benin; GSH, glutathione; MAL, Malawi; MW, molecular weight; UG, Uganda; vol, volume.

To identify the structural changes responsible for the higher activity in the resistant *GSTe2* Benin allele (119 F), its X-ray three-dimensional structure was determined and analyzed in comparison with that of a L119-GSTe2 susceptible allele cloned from Uganda (UG-GSTe2) (Figure 5A,B). The topology of GSTe2 indicates that the 221 amino acids of the *An. funestus* GSTe2 (afGSTe2) are divided into two distinct domains similar to that of *An. gambiae*[27] (92% similarity between the two species, with a difference of 20 amino acids). The N-terminal domain and a C–terminal domain are connected by a short hinge loop called the linker (amino acids 80–89) (Figure 5C). The N-terminal domain (1–79) has the typical TRX-fold (βαβαββα) domain in which the central four-stranded mixed β-sheet (B1-B4) is flanked on one side by helices H1 and H3 and on the other side by H2. The C-terminal domain (90–221) is a five α-helix bundle (H4-H8) in which the long α-helix H4 is noticeably bent [27]. The active site is located in a deep cleft formed at the interface of the two domains, specifically by the interaction of H1 and H3 from the N-terminal domain and H4, H6 and H8 from the C-terminal domain.

**Figure 6:**
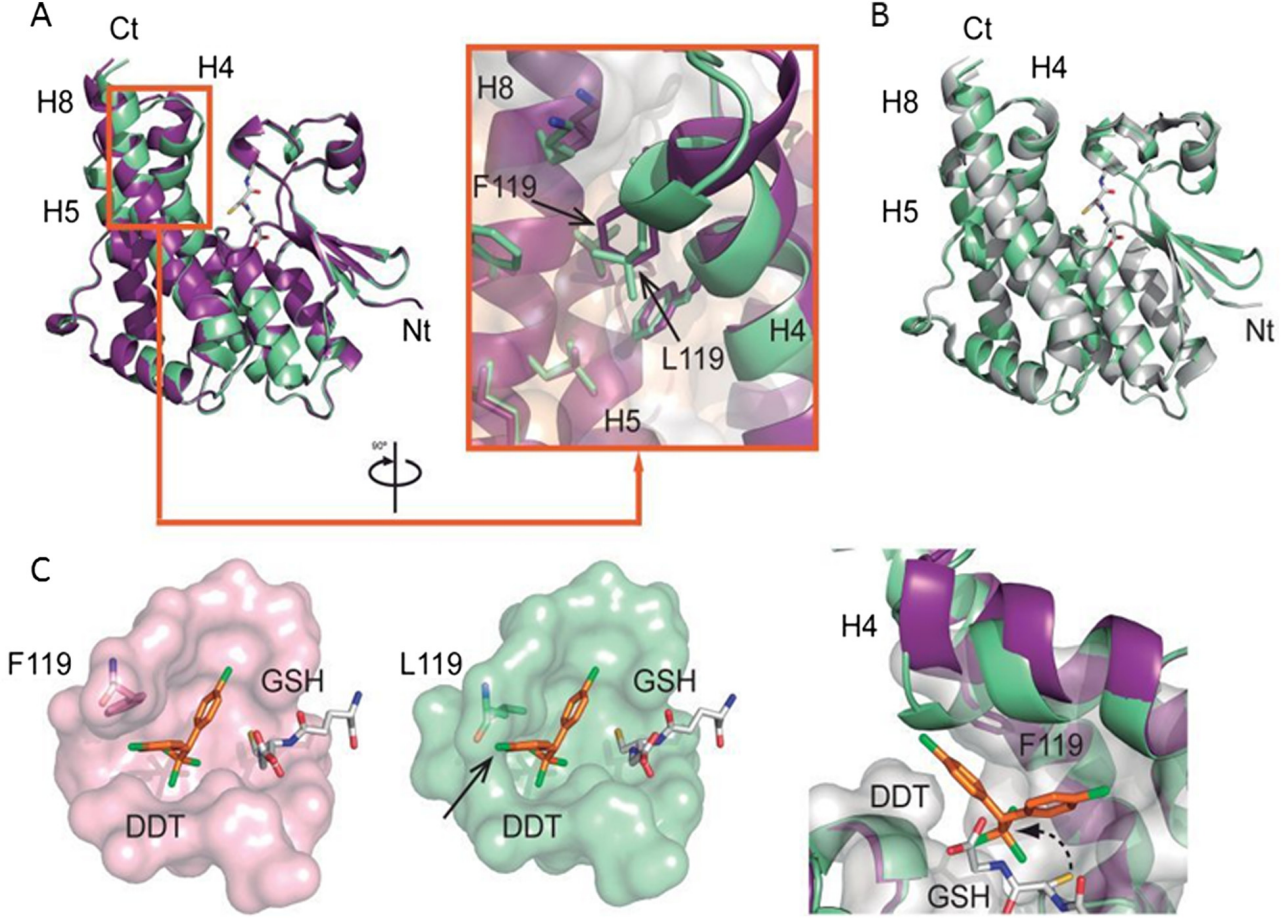
**GSTe2 three-dimensional structure variations reveal that L119F is important for DDT resistance. (A)** Overlay of Benin-GSTe2 (purple) and Uganda-GSTe2 (green) structures and a close-up of the H4/H8 helices containing the L119F mutation. **(B)** Overlay of Uganda-GSTe2 (green) and *An. gambiae* GSTe2 (grey) structures showing their very similar conformation. **(C)** Comparison of the substrate-binding pockets of Benin-GSTe2 (purple) and Uganda-GSTe2 (green). (Left) The molecular surface representation of the H-site shows that DDT docks well into the Benin-GSTe2 binding site but clashes with the Uganda-GSTe2 structure. (Right) Close-up of the effect of L119F on the H-site (this mutation bends H4, leading to an increase in the DDT-binding cavity in Benin-GSTe2 but not for Uganda-GSTe2). Ct, C-terminal; DDT, dichlorodiphenyltrichloroethane; GSH, glutathione; Nt, N-terminal.

Overall, the structure of BN-GSTe2 and UG-GSTe2 are very similar (Figure 5C, Additional file 11: Figure S6). However, significant local differences can be observed between the alleles at the C-terminal ends of H4 (containing the L119F mutation; residues from 113 to 128) and H8 (residues from 213 to 220) (Figure 6A). These differences can be described as a concerted movement of the BN-GSTe2 H4 and H8 helices with respect to those of UG-GSTe2, which opens the active site cleft. The root mean squared deviation (RMSD) between the structures is 0.64 Å for all Cα atoms. There is a significant decrease of the RMSD between the alleles from 0.64 to 0.42 Å when excluding H4 (containing the 119 F mutation) and the RMSD is only 0.33 Å when excluding both H4 and H8. A higher RMSD is observed when the two alleles are compared only for the H4 helix (RMSD = 0.9 Å) (Figure 6A). Furthermore, the significant difference between the BN-GSTe2 and UG-GSTe2 alleles is further compounded by the observation that the UG-GSTe2 allele structure is more similar to the structure of the *An. gambiae* GSTe2 protein [PDB:2imi] (RMSD = 0.4 Å) than to Benin-GSTe2 (RMSD = 0.64 Å) despite a 20-amino-acid difference (92% similarity) (Figure 6B), indicating that the L119F mutation is the key factor that modifies the GSTe2 structure (Figure 6C).

The active site of GSTe2 can be divided into two sub-sites. Firstly, there is the glutathione binding site, called the G-site, where one GSH molecule is bound. Secondly, there is the H-site, which recognizes the hydrophobic substrate. The architecture of the G-site of this superfamily of proteins is well described and nearly identical to that observed in BN-GSTe2 and UG-GSTe2. Although slight variations were observed between the G-sites of both alleles, there is no significant conformational difference, and these G-binding pockets do not have any major rearrangement (Additional file 11: Figure S6). The H-site is a large and slightly open hydrophobic cavity adjacent to the G-site. It is built of residues from different loops at the N-terminal domain and noticeably includes the variable C-terminal ends of helices H4 and H8 at the C-terminal domain (Figure 5C). Consequently, the differences observed between BN-GSTe2 and UG-GSTe2 mainly affect the structure of the substrate-binding pocket (H-site) (Figure 6A).

#### Putative DDT-binding pocket (H-site) and the DDT metabolism reaction

Leu119 normally forms part of the solvent-inaccessible hydrophobic core that stabilizes the conformation of helices H4, H5 and H8 of Uganda-GSTe2 (Figure 6A). However, to accommodate the bulkier Phe119 side chain in the Benin-GSTe2 structure, the N-terminal end of H4 has a dramatic bend, which increases the size of the H-site in comparison to the UG allele, where the cavity is tighter (Figure 6C). The H-site surface representation of both proteins with a bound GSH confirms that the resistant BN allele has a larger putative DDT cavity than the susceptible UG allele (Figure 6C).

Attempts to co-crystallize the substrate DDT or the product DDE with BN-GSTe2 and UG-GSTe2 were unsuccessful. Therefore, to investigate the substrate-binding properties of both alleles and assess their capacity to hydrolyze DDT, a substrate molecule was docked into the H-site (Figure 6C). This docking of a DDT molecule into the H-site of both alleles demonstrated that the Benin-GSTe2 has the appropriate size and shape to accommodate DDT better into a ‘close to reactive’ conformation. This includes the proper stabilization of the DDT chloride-phenyl rings and the optimal positioning of the DDT Cα, pointing towards the thiolate from glutathione (Figure 6C) leading to increased DDT metabolism. In contrast, the smaller H-site of Uganda-GSTe2 does not allow DDT recognition in this conformation (Figure 6C) leading to reduced DDT access and metabolism. This difference at the H-site explains the increased active site accessibility, the higher activity and consequently the high DDT resistance of the Benin population compared with other populations such as those from Uganda or Malawi.

A comparison of the structure of GSTe2 for *An. funestus* to the structures of AgGSTe2 and agGSTd1-6 clearly indicates that the key factors in the high metabolic activity of BN-GSTe2 are the H4 helix position and the shape and size of the DDT-binding cavity (Additional file 12: Figure S7). *GSTd1-6*, which belongs to the delta class of GST, possesses less than 1/350 the DDT-metabolizing activity of *AgGSTe2*[7] and was previously shown to differ from AgGSTe2 mainly at the H4 and H8 helices [27]. The H-site surface representation of these four proteins shows that the *AgGSTd1-6* cavity, although larger than those of *AfGSTe2s* and *AgGSTe2*, does not have the proper shape for docking a DDT molecule inside. This explains why it has the lowest DDT-metabolizing activity. BN-GSTe2, UG-GSTe2 and agGSTe2 have a V-shaped cavity, which will fit DDT. However, BN-GSTe2 has the largest binding pocket, which is able to accommodate the DDT molecule in a ‘close to reactive’ conformation leading to the higher resistance it confers to DDT.

The proteins for the BN-*GSTe2* and UG-*GSTe2* alleles are separated by two amino acid changes, L119F and I131V. However, only L119F is located in the H4 helix whereas I131V is located in the H5 helix, where no conformational change is observed between the BN and UG forms. In addition, the AgGSTe2 form, which is structurally similar to UG-GSTe2, contains I131, like the BN form. Therefore, this indicates that the I131V mutation does not play a role in the DDT metabolic activity of the BN allele and confirms that the molecular basis of the high DDT resistance conferred by the BN-GSTe2 allele compared with the susceptible allele is solely explained by the significant conformational changes that are induced by the L119F mutation. This finding is in agreement with what was previously predicted by [27]. This previous study suggested that for the *An. gambiae* GSTe2 to be more active against DDT, more conformational changes in the DDT-binding pocket were needed to further accommodate a DDT substrate and that such adjustments should not be on a large scale because the pocket was already well shaped. These changes are exactly what happened in the Benin form, where a single amino acid change induced the adjustment needed to accommodate more DDT in this resistant *An. funestus* strain, conferring a high level of DDT resistance in contrast with the UG allele, where this adjustment is absent. Overall, these structural analyses demonstrate that the L119F mutation is the causative mutation that confers DDT resistance.

## Conclusions

This study presents a comprehensive and detailed dissection of the genetic, molecular and structural basis of the metabolic resistance to an insecticide in a major malaria vector. Firstly, we conclusively detected the main gene responsible and showed that the resistance is conferred by a qualitative and a quantitative change in the DDT metabolizing enzyme (GSTe2). Secondly, we detected, for the first time, to our knowledge, for a mosquito species, a molecular resistance marker for metabolic resistance and designed a reliable DNA-based diagnostic assay that can accurately detect and map the distribution of resistance across Africa. This diagnostic tool will be valuable for vector control programs as resistance can be detected at an early stage, allowing suitable resistance management strategies to be implemented. Thirdly, we showed that the geographical distribution of DDT resistance, its origin and future spread patterns can be established or predicted based on the patterns of *GSTe2* genetic diversity, which are mainly due to the single L119F mutation.

## Materials and methods

### Mosquito collection

Blood-fed adult female *An. funestus* mosquitoes resting indoors were collected in houses between 6.00 AM and 12.00 PM in Pahou (6°23′N, 2°13′E), which is located near the Atlantic coast in southern Benin, west Africa. Several collections were conducted between July 2009 and April 2011. Another collection was conducted in Kpome (6°55′N, 2°19′E), which is located inland approximately 100 km from Pahou, in December 2011. The other samples used in this study have been previously described, and the respective references are provided when the samples are mentioned. Mosquito collection and rearing were performed as described previously [6,9]. Briefly, F_1_ adults were generated from field-collected female mosquitoes (using an egg-forced laying method [9]) and were randomly mixed in cages for subsequent experiments. All females used for individual oviposition were morphologically and molecularly identified as *An. funestus* ss, as previously described [21].

### Bioassays

Insecticide susceptibility assays with 4% DDT and 0.75% permethrin were conducted using 2- to 5-day-old F_1_ adults from pooled F_1_ mosquitoes, as described previously [21].

### Microarray

A custom *An. funestus* microarray chip containing 44,000 probes (60-mer) (A-MEXP-2245), previously described by Riveron *et al*. [5], was used to identify the set of genes associated with DDT resistance in Benin mosquitoes. Labeled complementary RNA (cRNA) was obtained from three biological replicates of DDT-resistant mosquitoes from Pahou that had been unexposed to insecticide and from susceptible unexposed mosquitoes (from the fully susceptible laboratory strain FANG). The Pahou mosquitoes were all DDT resistant as no mortality was recorded after 1 hr exposure to 4% DDT [6].

RNA was extracted from three batches of ten *An. funestus* females that were all 2 to 5 days old from the two sample sets using the Picopure RNA isolation kit (Arcturus, Applied Biosystems, Carlsbad, CA, USA). The quantity and quality of extracted RNA were assessed using the NanoDrop ND1000 spectrophotometer (Thermo Fisher, Waltham, MA, US) and Bioanalyzer (Agilent, Santa Clara, CA, USA), respectively. The cRNA from each sample was amplified using the Agilent Quick Amp labelling Kit (two-color) following the manufacturer’s protocol. The cRNA from the resistant Pahou samples was labelled with cy5 dye whereas the susceptible strain FANG (S) was labelled with the cy3 dye. The cRNA quantity and quality were assessed before labelling using the NanoDrop and the Bioanalyzer. Labelled cRNAs were hybridized to the arrays for 17 hr at 65°C, according to the manufacturer’s protocol. Five hybridizations were conducted by swapping the biological replicates.

Microarray data were analyzed using Genespring GX 12.0 software. To identify the differentially expressed genes, a twofold-change cutoff and a significance level of *P* < 0.01 with Benjamin-Hochberg correction for multiple testing were applied.

### Quantitative RT-PCR

The genes that were most associated with resistance from the microarray analysis were assessed by qRT-PCR to validate their expression pattern using three biological replicates for Pahou resistant mosquitoes (R) (alive after 24 hr exposure to DDT), Pahou control mosquitoes (C) (not exposed to any insecticide) and susceptible FANG mosquitoes (S). The primers are listed in Additional file 13: Table S5. Next, 1 μg total RNA from each of the three biological replicates from Pahou resistant, Pahou control and susceptible FANG mosquitoes was used as the template for cDNA synthesis using Superscript III (Invitrogen, Carlsbad, CA, US) with oligo-dT20 and RNase H, according to the manufacturer’s instructions. The qRT-PCR amplification was conducted as previously described [5]. The relative expression and fold-change of each target gene in R and C relative to S was calculated according to the 2^-ΔΔCT^ method, incorporating PCR efficiency [28] after normalization with the housekeeping genes *RSP7* (ribosomal protein S7, AGAP010592) and actin 5C (AGAP000651).

### Transgenic expression of *GSTe2* in *Drosophila* flies

#### Construction of transgenic *Drosophila* lines

The full-length *GSTe2* gene was amplified from the cDNA of the resistant Benin mosquitoes using Phusion High-Fidelity DNA Polymerase (Fermentas, Burlington, Ontario, Canada) and the following conditions: 1 cycle at 95°C for 5 min; 35 cycles of 94°C for 20 s, 57°C for 30 s and 72°C for 60 s; and 1 cycle at 72°C for 5 min. The primers used are listed in Additional file 13: Table S5. The PCR products were purified using the QIAquick PCR Purification Kit (Qiagen, Valencia, CA, USA) and cloned into the pJET1.2/blunt cloning vector using the CloneJET^TM^ PCR Cloning Kit (Fermentas, Burlington, Ontario, Canada). Five positive clones from both samples were purified with a QIAprep® Miniprep kit (Qiagen, Valencia, CA, USA) and sequenced on both strands. After sequence analysis, one clone of the *GSTe2* that was predominant in Benin mosquitoes was selected for constructing the transgenic flies. This clone was re-amplified as described above with primers containing restriction sites for *Bgl*II and *Xba*I. The purified PCR products were digested using the *Bgl*II and *Xba*I enzymes (Fermentas, Burlington, Ontario, Canada) , cloned into the pUASattB vector (provided by Dr J Bischof, University of Zurich), pre-digested with the same restriction enzymes and transformed into JM109 cells (Promega, Madison, Wi, US). Using the PhiC31 system, clones were injected into the germ-line of *D. melanogaster* carrying the attP40 docking site on chromosome 2 (y^1^ w^67c23^; P (CaryP) attP40,1;2) [29]. One transgenic line, UAS-GSTe2, was generated and balanced. For the expression of the transgene *GSTe2*, ubiquitous expression was obtained in the flies using the Act5C-GAL4 strain (y^1^ w^*^; P (Act5C-GAL4-w) E1/CyO,1;2) (Bloomington Stock Center, IN, USA).

#### Confirmation of GSTe2 expression in the transgenic flies by quantitative RT-PCR

To confirm the expression of *GSTe2* in the experimental groups and the absence of expression in the control groups, total RNA was extracted from three pools of five flies. The cDNA synthesis was conducted as described above. PCR was performed using the synthesized cDNA as a template and primers specific to *GSTe2* (Additional file 13: Table S5). In addition, the relative expression levels of the *GSTe2* transgene were assessed by qRT-PCR in the F_1_ progeny under the Act5C driver and in respective controls with normalization using the *RPL11* housekeeping gene.

#### Drosophila contact bioassays

Females from F_1_ expressing *GSTe2* were selected as the experimental group for the insecticide bioassays. The parental lines and the female progeny from the cross between the Act5C-GAL4 females and males that did not carry the *GSTe2* transgene (but with the same attP40 background) were used as controls. A comparison of the mortality rates between the experimental groups and the control groups was used to assess whether *GSTe2* was conferring resistance. In addition, 2- to 5-day-old females post-eclosion were used in a contact assay with 4% DDT and the pyrethroid insecticides permethrin (2%) and deltamethrin (0.15%), as described previously [5]. Then 20 to 25 flies were placed in each vial, and the mortality plus knockdown was scored after 1 hr, 2 hr, 3 hr, 6 hr, 12 hr and 24 hr of exposure to the insecticide. For all assays, at least six replicates were performed. Student’s *t*-test was used to compare the mortality plus knockdown in the experimental group with each control group.

### Analyzing *GSTe2* polymorphisms in relation to DDT resistance

#### Analysis of cDNA polymorphisms

The full-length cDNA of *GSTe2* was cloned and sequenced for DDT-resistant mosquitoes from Benin and for DDT-susceptible *An. funestus* across Africa (Uganda, Malawi, Mozambique and Zambia) to detect potential mutations that could be associated with DDT resistance. The cDNA amplification was performed using the same cDNA synthesized for qRT-PCR with the Phusion polymerase, and the product was cloned and sequenced as described above. The primers used are listed in Additional file 13: Table S5.

#### Polymorphism analysis between susceptible and resistant field mosquitoes in Benin

A further assessment of the correlation of the polymorphism of *GSTe2* and DDT resistance was conducted by individually amplifying and direct-sequencing the genomic full-length sequence of *GSTe2* (all exons and introns) for six susceptible mosquitoes (dead after 1 hr exposure) and six resistant mosquitoes (alive after 1 hr exposure) from Kpome because no susceptible mosquitoes were obtained in Pahou. The polymorphic positions were detected through a manual analysis of sequence traces using BioEdit and as sequence differences in multiple alignments using ClustalW [30]. dnaSP 5.10 [31] was used to define the haplotype phase (through the Phase option) and to assess genetic parameters, such as nucleotide diversity π, haplotype diversity and the D and D* selection estimates. A maximum likelihood tree of the haplotypes for both cDNA and genomic amplifications was constructed using MEGA 5.2 [32], and a haplotype network was built using the TCS program [33] (95% connection limit, gaps treated as a fifth state) to assess the potential connection between haplotypes and resistance phenotypes.

### Diagnostic assay and assessment of whether L119F correlates with DDT resistance

To genotype the *GSTe2* L119F mutation in field populations of *An. funestus*, a custom TaqMan assay was designed after repeated failures from pyrosequencing due to the many thymine nucleotides (Ts) around the C/T mutation site. The primer and reporter sequences are provided in Additional file 14: Table S6. The TaqMan reactions were performed in a 10-μl final volume containing 1× SensiMix (Bioline, London, UK), 800 nM of each primer and 200 nM of each probe using an Agilent MX3005P machine. The following cycling conditions were used: 10 min at 95°C, 40 cycles of 15 s at 92°C and 1 min at 60°C. This assay was used to assess the correlation between the genotypes of the L119F mutation and DDT-resistant phenotypes. For this assay, due to the lack of susceptible mosquitoes from Pahou and the few susceptible mosquitoes from Kpome (in Benin), *An. funestus* samples were collected in northern Cameroon from Gounougou (9°05′N, 13°40′E), as described above. A WHO bioassay for DDT was conducted as described above. Then 35 mosquitoes that were dead after 1 hr of 4% DDT exposure (susceptible) and 35 alive mosquitoes (resistant) from Gounougou were genotyped for the L119F mutation using the TaqMan assay.

#### L119F correlation with pyrethroid resistance

To assess the correlation between the genotypes of the L119F mutation and pyrethroid-resistant phenotypes, 25 mosquitoes that were dead after 1 hr of 0.75% permethrin (type I pyrethroid) exposure (susceptible) and 25 alive mosquitoes (resistant) from Gounougou were genotyped for the L119F mutation using the TaqMan assay. The same genotyping was conducted for lambda-cyhalothrin (a type II pyrethroid). Association between resistance phenotypes and the genotypes was assessed by estimating the odds ratios and the statistical significance based on the Fisher exact probability test [34].

#### Contribution of *GSTe2* upregulation and the 119 F mutation to the resistance phenotype

To assess whether *GSTe2* upregulation and the presence of the 119 F mutation are both necessary to confer DDT resistance, we compared the expression levels of *GSTe2* for the three genotypes of the L119F mutation alongside the susceptible FANG strain using qRT-PCR. Three batches of five mosquitoes from Gounougou were used for homozygote susceptible samples (C/C; L119/L119), heterozygote samples (C/T; L119/119 F) and homozygote resistant samples (T/T; 119 F/119 F) following the protocol described above. The genotype of each mosquito was first established after a TaqMan assay using gDNA (genomic DNA) extracted from the legs, and then mosquitoes from the same genotype were pooled for RNA extraction and cDNA synthesis.

### Geographical distribution of the *L119F* mutation across Africa

To assess the geographical distribution of the L119F mutation across Africa, 30 field-collected *An. funestus* ss females from nine countries belonging to different regions of Africa were genotyped by TaqMan: Benin (Pahou, collected in 2010–2011), Ghana (Obuasi, 2009), Burkina (Bobo-Dioualasso, 2010) and Cameroon (Gounougou, 2006) in west-central Africa; Uganda (Tororo, 2009) and Kenya (Kisumu, 2012) in east Africa; and Malawi (Chikwawa, 2009), Zambia (Katete district, 2011) and Mozambique (Chokwe, 2009) in southern Africa.

### Population structure of *GSTe2* across African *An. funestus* populations

To assess the patterns of genetic variability of *GSTe2* across African populations of *An. funestus* in relation to DDT resistance and to detect the potential signatures of selection on this gene, full-length *GSTe2* (exons and introns) was amplified and directly sequenced for 10–15 field-collected female mosquitoes from six countries from different regions of Africa. These countries are Benin, Ghana and Cameroon in west-central Africa, Uganda in east Africa and Mozambique and Malawi in southern Africa. The patterns of genetic variability were analyzed as described above using dnaSP 5.10 [31]. The levels of pairwise genetic differentiation between the populations were estimated in dnaSP 5.10 using the *K*_*st*_ statistic [35], although *F*_*st*_ and *N*_*st*_ estimates were also obtained for comparison. The significance of the *K*_st_*estimates was assessed by permutation of subpopulation identities and re-calculating *K*_st_* 10,000 times, as implemented in dnaSP 5.10.

### Phylogenetic tree of *GSTe2* haplotypes

A maximum likelihood phylogenetic tree for the coding sequences of *GSTe2* across Africa was constructed using MEGA 5.2 [32]. The best-fit substitution model was firstly assessed based on the Bayesian information criterion. This indicated that the Jukes–Cantor model best describes the *GSTe2* haplotype dataset out of 24 candidate models. The Jukes–Cantor model was then used to generate the maximum likelihood tree as implemented in MEGA with 500 bootstrap replications to assess the robustness of the tree. Additionally, a haplotype network was built for both the full-length region (non-coding plus coding) and the coding region only, using the TCS program [33] (95% connection limit, gaps treated as a fifth state), to assess the potential connection between haplotypes and resistance phenotypes.

#### Test of selection on *GSTe2*

To test for positive selection acting on *GSTe2* in relation to DDT resistance, several tests were carried out. Because reduced levels of genetic variation are an indication of positive selection particularly when there is a sweep through population nearing fixation [24], several genetic diversity estimates were computed using dnaSP 5.10 and compared between the six populations. These parameters included the nucleotide diversity (*π*), the haplotype diversity and *θ*, an estimate of 4*N*_*e*_*μ*, with *N*_*e*_ the effective population size and *μ* the mutation rate per nucleotide. Standard deviation estimates were computed by dnaSP 5.10.

Departure from neutrality was also tested using different selection tests such as the HKA test and the MK test as implemented in dnaSP 5.10 using the *GSTe2* sequence from *An. gambiae* (AGAP009194) for the out-group. Possible evidence of positive selection was also investigated using the *K*_*a*_/*K*_*s*_ ratio (non-synonymous substitution rate/synonymous substitution rate) with *K*_*a*_ / *K*_*s*_ > 1 indicating positive selection, *K*_*a*_ / *K*_*s*_ < 1 implying purifying selection and *K*_*a*_ / *K*_*s*_ = 1 suggesting neutrality [36]. Additionally, a codon-based Z test of selection was carried out to assess further the signature of positive selection of *GSTe2* in resistant samples. This test uses the Nei and Gojobori method to compute the numbers of synonymous (*dS*) and non-synonymous (*dN*) substitutions per site and the numbers of potentially synonymous and potentially non-synonymous sites [37] as implemented in MEGA 5.2. The *dN*/*dS* ratio was calculated and the probability of rejecting the null hypothesis of strict neutrality (H0: *dN* = *dS*) in favor of the alternative positive selection hypothesis (H1: *dN* > *dS*) was estimated using the bootstrap method (1,000 replicates) in MEGA 5.2.

### GSTe2 protein expression and purification

A resistant *GSTe2* allele (119 F-GSTe2) from Benin (BN) and two susceptible alleles (L119-GSTe2) from Uganda (UG) and Malawi (MAL) were cloned into a pET28a expression plasmid between *Nde*I and *Xho*I sites to yield pET28a::BN-GSTe2, pET28a::UG-GSTe2 and pET28a::MAL-GSTe2 constructs. The pET28a::BN-GSTe2, pET28a::UG-GSTe2 and pET28a::MAL-GSTe2 plasmids were transformed into *Escherichia coli* strain BL21 (DE3) (Novagen, Madison, WI, US) for protein expression using standard protocols. A total of 5 ml of an overnight culture was sub-cultured into 500 ml of fresh 2TY broth medium plus kanamycin (50 μg/ml). The transformed cells were grown at 37°C. *GSTe2* expression was then induced with 0.3 mM of isopropyl-β-D-thiogalactoside when the OD (optical density) at 600 nm was 0.6 to 0.8 overnight at 16°C. The cells were harvested by centrifugation (15 min, 4,500 *g*); resuspended in 25 mM TrisHCl pH 8.0, 500 mM NaCl, 20 mM imidazole and 5 mM β-mercaptoethanol; and disrupted by sonication. After centrifugation (40 min, 40,000 *g*), the clear supernatant was filtered, and the His-tagged GSTe2s was purified using Ni-NTA agarose (Qiagen,Valencia, CA, US) according to the manufacturer’s instructions. The filtered supernatant was mixed with the previously equilibrated beads. After incubation, a washing step with ten volumes of 25 mM TrisHCl pH 8.0, 500 mM NaCl, 20 mM imidazole and 5 mM β-mercaptoethanol buffer was performed. All constructs yielded 26.8 kDa products. After a full dialysis against 25 mM TrisHCl pH 8.0, 200 mM NaCl and 5 mM β-mercaptoethanol, the His-tag was cleaved using 7.5 units of thrombin per mg of tagged protein. A final purification step was performed using a Superdex 200 16/60 column (Amersham Biosciences Limited, London, UK) to obtain a highly purified sample (Figure 5A). The time courses of the chromatography were monitored by SDS-PAGE (Figure 5B). GSTe2 proteins were concentrated to 23 mg/ml with a 10-kDa cutoff Amicon protein concentrator (YM-10; Millipore Corporation, Bedford, MA, USA). The final protein concentration was determined spectrophotometrically using the calculated molar absorption coefficient at 280 nm. The samples were kept at 4°C.

#### Metabolic assay to assess the effect of *GSTe2* on DDT, permethrin and deltamethrin

The activity of GST was determined with a spectrophotometric assay to examine the formation of the conjugate of 1-chloro-2,4-dinitrobenzene (CDNB) and reduced GSH. One unit of enzyme is defined as the amount of enzyme that yields 1.0 μmol of conjugate product per minute at pH 6.5 and 30°C. Metabolism assays were conducted at 30°C for 60 min with shaking at 1,200 rpm, in a total volume of 0.5 ml. The buffer system was 0.1 M potassium phosphate buffer (pH 6.5), 2.5 mM GSH and 0.2 units of enzyme in the presence of 10 μg/ml DDT, 0.025 mg/ml permethrin or 0.03 mg/ml deltamethrin all in methanol (the solvent did not exceed 10% of the reaction total volume). The control sample contained the same reagent mixture with the boiled recombinant enzyme. After 1 hr of incubation, 500 μl of methanol was added to stop the reaction and the samples were then centrifuged at 13,000 rpm for 20 min at room temperature and 200 μl of the resulting supernatant were transferred to HPLC vials. The quantity of DDT, DDE and pyrethroid remaining in the samples was determined by reverse-phase HPLC with a monitoring absorbance wavelength of 232 nm (Chromeleon, Dionex, Sunnyvale, CA, US). Briefly, 100 μl of sample was injected into a 250 mm C18 column (Acclaim 120, Dionex, Sunnyvale, CA, US) at 23°C. Separation of DDT and DDE was achieved using an isocratic mobile phase of 92% methanol and 8% water with a flow rate of 1 ml/min. Kinetic studies were conducted as previously described [8]. The results were analyzed by non-linear regression using GraphPad Prism v4.0 software (GraphPad Software, Inc, San Diego, CA, USA).

#### Additional *GSTe2* metabolism of permethrin by gradient run

To better resolve the metabolite peaks observed for permethrin with the initial isocratic run, a further analysis of the metabolism profile of permethrin by GSTe2 was carried out using a gradient run condition. The parent compound and its metabolites were separated on an C18 Acclaim column by injecting 100 μl of reaction products reconstituted in 1 ml methanol after an ethyl acetate extraction and evaporation step. The reaction mixture was 2 ml with 10 μg/ml permethrin and 0.2 unit of *GSTe2*. The reaction was initiated by adding 2.5 mM GSH. A bovine serum incubation mixture was used as a negative control. The mobile phases used were the solvent acetonitrile (A), methanol (B) and H_2_O (C). The analytes were eluted with the following gradient programs (linear increase): 0 min (0% A, 5% B, 95% C), 15 min (37% A, 5% B, 58% C), 25 min (60% A, 5% B, 35% C), 50 min (85% A, 5% B, 10% C), 51 min (95% A, 5% B, 0% C), 56 min (95% A, 5% B, 0% C), 61 min (0% A, 5% B, 95% C) and 69 min (0% A, 5% B, 95% C), at a flow rate of 1 ml/min. Peaks were detected at 232 nm. Data collection and analysis were conducted using Chromeleon software.

### Determination of *GSTe2* structure and docking of DDT alleles

#### Crystallization

GSTe2 from Benin, Uganda and Malawi mosquitoes was purified as described in the supplementary information for the metabolic assays. The GSTe2 initial crystallization conditions were investigated by high-throughput techniques with a NanoDrop robot (Innovadyne Technologies Inc., Santa Rosa, CA, US ) using the commercial screen solutions Crystal Screen 1 and 2 (Hampton Research, Aliso Viejo, CA, US), PACT Suite and JCSG Suite (Qiagen,Valencia, CA, US). Crystallization assays were conducted using the sitting-drop vapor-diffusion method at 18°C in 96-well plates (Innovaplate SD-2 microplates, Innovadyne Technologies Inc., Santa Rosa, CA, US ). Drops of 250 nl protein at 23 mg/ml and 250 nl precipitant solution were mixed and equilibrated against 65 μl of the well solution. Preliminary crystallization conditions led to crystal clusters of thin plates. Several strategies were used to optimize the best crystallization conditions, which included adjusting the protein sample composition, the precipitant concentration and pH values, and screening with different additives (Additive Screen, Hampton Research, Aliso Viejo, CA, US) or detergents. The final conditions were scaled up on 24-well plates (Linbro plates, Hampton Research, Aliso Viejo, CA, US) through hanging-drop experiments and on a 60-well microbath under oil (Terasaki plates) at 18°C.

The crystals used in our analysis grew from a mix of 1 μl of the protein solution at 23 mg/ml and 2 μl of a precipitant solution. After the refinement of several parameters, isolated prismatic and rod-shaped crystals were obtained. BN-GSTe2 was crystallized by hanging-drop vapor-diffusion using a solution as precipitant containing 40% (v/v) PEG 300 and 0.1 M phosphate-citrate pH 4.2. However, UG-GSTe2 and MAL-GSTe2 were crystallized using a microbatch under oil technique with precipitant 25% w/v PEG 1500 and 0.1 M PCB (Propionate, Cacodylate, Bis-Tris Propane) buffer pH 6.0, and 25% w/v PEG 1500 and 0.1 M MMT (L-Malic acid, MES, Tris)buffer pH 5.0, respectively. To grow holo_GSTe2 crystals, 0.5 μl of 10 mM GSH (L-glutathione reduced)/ GSSG (L-glutathione oxidized) from Hampton Research were added to the crystallization drops. Several cryoprotectants were tested, including glycerol, MPD (2-Methyl-2,4-pentanediol) and polyethylene glycol. The best cryoprotectant solution was 20% glycerol on the crystal mother liquor.

#### Data collection and structure resolution

Crystals were mounted on a fiber loop, transferred to the cryoprotectant solution and flash-frozen at 100 K in a nitrogen gas steam. Preliminary diffraction data were collected using an in-house Imaging Plate Mar345dtb detector (MarResearch, Norderstedt, Germany) with Cu K_α_ X-rays generated by a rotating-anode generator (MicroStar, Bruker, Billerica, MA, US) with Helios mirrors (Bruker, Billerica, MA, US) operated at 45 kV and 60 mA. The apo_UG-GSTe2 and holo_MAL-GSTe2 crystals were not suitable for X-ray data analysis because their diffraction was very poor. However, the crystals of apo_BN-GSTe2, holo_BN-GSTe2 and holo_UG-GSTe2 had good diffraction patterns. A complete diffraction dataset was collected for each using the European Synchrotron Radiation Facility (Grenoble, France) (see details in Additional file 15: Table S7). Diffraction data were processed with XDS (X-ray Detector Software) [38] and scaled with SCALA from the CCP4 package (Collaborative Computational Project, Number 4, 1994). Molecular replacement with the program Phaser [39] was used to resolve the GSTe2 structures. The coordinates from the *An. gambiae* glutathione S-transferase epsilon 2 (agGSTe2) [PDB:2IL3] (92% sequence identity [27]) were used to resolve the apo_BN-GSTe2 and holo_UG-GSTe2 structures. The holo_BN-GSTe2 structure was resolved using the holo_UG-GSTe2 coordinates. Several cycles of restrained refinement with PHENIX [40] and iterative model building with COOT were required to obtain the final models. The water molecules were also modeled. The data collection, data processing and model refinement statistics are summarized in Additional file 15: Table S7.

The stereochemistry of the models was verified with MolProbity, and the molecular model figures were produced using PyMol (The PyMOL Molecular Graphics System, Version 1.5.0.4 Schrödinger, LLC, Portland, OR, US). The RMSDs between the proteins structures were calculated in COOT. The DALI algorithm was used for structure-based sequence alignment of BN-GSTe2 and UG-GSTe2 with other insect GSTs.

### Docking

Docking of DDT to the different alleles of GSTe2 was performed using the Genetic Optimization for Ligand Docking (GOLD) software from the Cambridge Crystallographic Data Center, UK. Both DDT and DDE were used in the docking calculations. The 3D structures of these molecules were obtained from the Cambridge Structural Database with reference codes CPTCEL and DCLPEY, respectively. The stereochemistry of the ligands was confirmed with the Mercury program. Both holo_BN-GSTe2 and holo_UG-GSTe2 coordinates were used to define the binding site for molecular docking studies. All the solvent molecules in the structures were removed, and hydrogen atoms were added to the whole protein. In addition, hydrogen atoms were added to the cofactor molecule and cysteine SH group was deprotonated to obtain GS^−^ for the docking calculations. The docking cavity was defined as a collection of amino acids enclosed within a sphere with a 10 Å radius around the GS^−^ molecule, giving freedom of movement to F118, R112, E116, L119 or F119, F120, T165 and L207 and more restrained flexibility to M111 and F115 in the side-chain rotamers. The standard default settings were used in all calculations (number of dockings: 10), but early termination was allowed when the top three solutions were within 1.5 Å RMSD from each other.

#### Data access

The DNA sequences reported in this paper have been deposited in the GenBank database [GenBank:KC800340-GenBank:KC800421], the microarray data in ArrayExpress (E-MTAB-1578) and the 3D X-ray structures in the PDB database (BN-GSTe2-GSH [PDB:3zmk] and UG-GSTe2-GSH [PDB:3zml]).

## Abbreviations

BN: Benin; bp: base pair; CAM: Cameroon; cRNA: complementary RNA; DDT: dichlorodiphenyltrichloroethane; FC: fold-change; GH: Ghana; GSH: glutathione; GST: glutathione S-transferase; hd: haploid diversity; HKA: Hudson, Kreitman and Aguade test; IRS: indoor residual spraying; kb: kilobase; kdr: knockdown resistance; LLIN: long lasting insecticide net; MAL: Malawi; MK: McDonald and Kreitman test; MOZ: Mozambique; PCR: polymerase chain reaction; PDB: Protein Data Bank; PEG: poly(ethylene) glycol; qRT-PCR: quantitative reverse transcriptase polymerase chain reaction; RDL: resistance to dieldrin; RMSD: root mean square deviation; UG: Uganda; UTR: untranslated region; WHO: World Health Organization.

## Competing interests

The authors declare that they have no competing interests.

## Authors’ contributions

CSW conceived, designed and coordinated the research. RD, BDM and CSW carried out the sample collection and bioassays. HI and CSW performed the transcription analyses and carried out the genetic variability analysis. JMR performed the transgenic expression in *Drosophila*. CY performed the crystallographic study with AA. HMI, SSI and JMR performed the metabolic assays of GSTe2. HR and JH contributed toward data analysis and significant insights. JMR, CY, HMI and CSW wrote the manuscript with contribution from all the authors. All authors read and approved the final manuscript.

## Supplementary Material

Additional file 1: Figure S1 Transcription profiling and functional analyses of GSTe2. **(A)** Volcano plot showing the differential expression pattern between DDT-resistant Benin mosquitoes and the susceptible FANG strain, with a twofold-change cutoff and *P* < 0.01. *GSTe2* is highlighted as one of the most upregulated genes in Benin mosquitoes. **(B)** qRT-PCR validation of microarray upregulation of the main detoxification genes that were differentially expressed between resistant and susceptible DDT samples. c738 is a short-chain dehydrogenase (combined_c738) that is upregulated according to the microarray. **(C)** The relative expression of the *GSTe2* transgene in the transgenic *D. melanogaster* Act5C-GSTe2 strain and the control sample with no transgene expression. The data shown are the mean ± standard error of the mean (*n* = 3). **(D)** Deltamethrin bioassay tests on transgenic Act5C-*GSTe2* flies (Exp-GSTe2) and control strains (two parental (UAS-GSTe2 and GAL4-Actin) and F_1_ progeny that do not express the *GSTe2* transgene (Cont-NO)). Click here for file

Additional file 2: Table S1 Downregulated genes in the DDT-resistant Benin population of *An. funestus.* Click here for file

Additional file 3: Table S2 Genetic variability parameters for *GSTe2* for resistant (alive) and susceptible (dead) mosquitoes fromKpome (Benin). Click here for file

Additional file 4 A PDF document containing supplemental results and discussion. Click here for file

Additional file 5: Figure S2 Detection of the 119 F *GSTe2* resistance allele in *An. funestus*. **(A)** The results of the TaqMan diagnostic assay for genotyping L119F, with three genotypes unambiguously identified (three clusters). **(B)** The genotype distribution of L119F alleles across nine countries in Africa shows there is a strong correlation between L119F genotypes and known patterns of DDT resistance. For example, the 119 F (T/T) genotype is fixed in the highly DDT-resistant Benin population but is completely absent in the fully susceptible southern African populations (Malawi, Mozambique and Zambia). **(C)** Assessment of the correlation between the L119F alleles and the permethrin (type I pyrethroid)-resistant phenotype. **(D)** Assessment of the correlation between the L119F alleles and the lambda-cyhalothrin (type II pyrethroid)-resistant phenotype. Click here for file

Additional file 6: Figure S3 Metabolic activity of GSTe2. **(A)** DDT metabolism by GSTe2 from Benin has a high peak for the DDE metabolite product. **(B)** There is a reduction in the peak for permethrin metabolism by 119 F-GSTe2 in the reaction with GSTe2 (blue) compared to the control (black) (three replicates) using isocratic conditions. The arrow indicates potential metabolites. **(C)** Additional permethrin metabolism by the Benin GSTe2 enzyme using gradient conditions. Blue peaks refer to the metabolism profile of active GSTe2 whereas black peaks refer to the bovine serum incubation mixture (negative control). The arrows indicate the three potential metabolites. The analytes were eluted with a linear increase gradient program. Click here for file

Additional file 7: Table S3 Selection parameters for *GSTe2* across Africa. Click here for file

Additional file 8: Figure S4 Polymorphism patterns of *GSTe2* in Africa. **(A)** Plot of genetic diversity parameters of GSTe2 across Africa that indicates there is strong directional selection of *GSTe2* in Benin mosquitoes. hd, haplotype diversity; π, nucleotide diversity. **(B)** Haplotypes of *GSTe2* (coding and non-coding) across six countries in Africa with contrasting DDT phenotypes. The polymorphic positions are indicated with numbers above the nucleotide, and the haplotypes are labeled from 1 to 39 with preceding initials from the country where the haplotype is predominant. An asterisk (*) shows that the haplotype was observed in other countries. *N* is the number of individuals who share the haplotype. **(C)** The same but only considering coding regions. **(D)** The same but only considering non-synonymous substitutions providing the different protein variants of GSTe2 across Africa. Click here for file

Additional file 9: Figure S5 Haplotype network for the full-length *GSTe2* (coding and non-coding regions) across Africa**.** The frequency of each haplotype is reflected by the size of its polygon. Each node represents a segregating mutation (the polymorphic position is given above the branches). The polygons in grey are resistant haplotypes that harbor the resistant 119 F allele whereas the haplotypes in white are susceptible. The mutational position of 499 is circled to indicate that this polymorphic position, which corresponds to L119F, is key to shaping the *GSTe2* haplotype distribution. Click here for file

Additional file 10: Table S4 Genetic differentiation using *K*_st_. Click here for file

Additional file 11: Figure S6 The conformations of the GSH binding pocket (G-site) of the resistant BN-GSTe2 allele (purple) and the susceptible UG-GSTe2 allele (green) have no significant differences. The GSH cofactor and the protein amino acids and water molecules hydrogen-bonded to it are represented in ball-and-stick mode. Click here for file

Additional file 12: Figure S7 Comparative analysis of the H-site structure of different GSTe2 alleles and agGSTd1-6. The size and shape of the site are key determinants of DDT metabolism capacity. (Top) The molecular surface representations of those residues constituting the H-site of Uganda-GSTe2 (green), Benin-GSTe2 (purple), agGSTe2 (white) and agGSTd1-6 (yellow). Oxygen and nitrogen atoms at the surface are depicted in red and blue, respectively. (Bottom) Overlays of the structures of Uganda-GSTe2 (green) with Benin-GSTe2 (purple), agGSTe2 (white) and agGSTd1-6 (yellow) showing that most differences are in the secondary structural elements related to the H-site. Click here for file

Additional file 13: Table S5 List of primers used in this study. Click here for file

Additional file 14: Table S6 Primers for TaqMan L119F *GSTe2* assay. Click here for file

Additional file 15: Table S7 Summary statistics for data collection, processing and model refinement. Click here for file
