# Supplementary material for: A single mutation in the GSTe2 gene allows tracking of metabolically based insecticide resistance in a major malaria vector
Source: Genome Biol. 2014 Feb 25;15(2):R27. doi: 10.1186/gb-2014-15-2-r27 (PMC4054843; doi:10.1186/gb-2014-15-2-r27)
Supplement: Additional file 1: Figure S1 — Transcription profiling and functional analyses of GSTe2. (A) Volcano plot showing the differential expression pattern between DDT-resistant Benin mosquitoes and the susceptible FANG strain, with a twofold-change cutoff and P < 0.01. GSTe2 is highlighted as one of the most upregulated genes in Benin mosquitoes. (B) qRT-PCR validation of microarray upregulation of the main detoxification genes that were differentially expressed between resistant and susceptible DDT samples. c738 is a short-chain dehydrogenase (combined_c738) that is upregulated according to the microarray. (C) The relative expression of the GSTe2 transgene in the transgenic D. melanogaster Act5C-GSTe2 strain and the control sample with no transgene expression. The data shown are the mean ± standard error of the mean (n = 3). (D) Deltamethrin bioassay tests on transgenic Act5C-GSTe2 flies (Exp-GSTe2) and control strains (two parental (UAS-GSTe2 and GAL4-Actin) and F1 progeny that do not express the GSTe2 transgene (Cont-NO)). [file gb-2014-15-2-r27-S1.doc]

**Additional file 1: Figure S1.** **Transcription profiling and functional analyses of GSTe2.** (A) Volcano plot showing the differential expression pattern between DDT-resistant Benin mosquitoes and the susceptible FANG strain, with a twofold-change cutoff and *P* < 0.01. *GSTe2* is highlighted as one of the most upregulated genes in Benin mosquitoes. (B) qRT-PCR validation of microarray upregulation of the main detoxification genes that were differentially expressed between resistant and susceptible DDT samples. c738 is a short-chain dehydrogenase (combined_c738) that is upregulated according to the microarray. (C) The relative expression of the *GSTe2* transgene in the transgenic D. melanogaster Act5C-GSTe2 strain and the control sample with no transgene expression. The data shown are the mean ± standard error of the mean (*n* = 3). (D) Deltamethrin bioassay tests on transgenic Act5C-*GSTe2* flies (Exp-GSTe2) and control strains (two parental (UAS-GSTe2 and GAL4-Actin) and F_1_ progeny that do not express the *GSTe2* transgene (Cont-NO)).
