# Supplementary material for: A single mutation in the GSTe2 gene allows tracking of metabolically based insecticide resistance in a major malaria vector
Source: Genome Biol. 2014 Feb 25;15(2):R27. doi: 10.1186/gb-2014-15-2-r27 (PMC4054843; doi:10.1186/gb-2014-15-2-r27)
Supplement: Additional file 2: Table S1 — Downregulated genes in the DDT-resistant Benin population of An. funestus. [file gb-2014-15-2-r27-S2.doc]

**Table S1: Down-regulated genes in DDT resistant Benin population of *An. funestus***

| **Probe Name** | **Gene Name** | **P value** | **Fold change** | **Log2FC** | **Description** |
| --- | --- | --- | --- | --- | --- |
| CUST_4355_PI406199772 | CD577188.1 | 0.0008 | 44.3 | -5.5 | cytochrome c oxidase subunit iii |
| CUST_3909_PI406199772 | CD577421.1 | 0.0004 | 26.4 | -4.7 | NADH dehydrogenase subunit 3 |
| CUST_4375_PI406199772 | CD577178.1 | 0.0030 | 26.3 | -4.7 | cytochrome c oxidase subunit iii |
| CUST_9140_PI406199769 | combined_c4631 | 0.0020 | 23.9 | -4.6 | AGAP009107-PA |
| CUST_3907_PI406199772 | CD577422.1 | 0.0009 | 23.4 | -4.5 | hypothetical protein Phum_PHUM624180 [Pediculushumanuscorporis] |
| CUST_9141_PI406199769 | combined_c4631 | 0.0021 | 21.6 | -4.4 | AGAP009107-PA |
| CUST_3794_PI406199769 | combined_c1916 | 0.0081 | 21.6 | -4.4 | NA |
| CUST_1815_PI406199769 | combined_c916 | 0.0003 | 21.6 | -4.4 | NA |
| CUST_1746_PI406199769 | combined_c882 | 0.0010 | 21.0 | -4.4 | NA |
| CUST_4377_PI406199772 | CD577177.1 | 0.0007 | 20.9 | -4.4 | cytochrome c oxidase subunit iii |
| CUST_3908_PI406199772 | CD577422.1 | 0.0003 | 20.8 | -4.4 | hypothetical protein Phum_PHUM624180 [Pediculushumanuscorporis] |
| CUST_4334_PI406199772 | CD577200.1 | 0.0004 | 20.6 | -4.4 | cytochrome c oxidase subunit iii |
| CUST_4236_PI406199772 | CD577249.1 | 0.0005 | 20.4 | -4.4 | cytochrome b |
| CUST_4216_PI406199772 | CD577259.1 | 0.0016 | 19.8 | -4.3 | cytochrome b |
| CUST_3795_PI406199769 | combined_c1916 | 0.0029 | 19.0 | -4.2 | NA |
| CUST_6652_PI406199769 | combined_c3364 | 0.0008 | 18.3 | -4.2 | AGAP009859-PA [Anopheles gambiae] |
| CUST_3710_PI406199769 | combined_c1873 | 0.0005 | 18.3 | -4.2 | monkey king protein |
| CUST_3053_PI406199772 | CD577873.1 | 0.0003 | 15.3 | -3.9 | pyruvate dehydrogenase e1 component  subunit alpha |
| CUST_2388_PI406199769 | combined_c1205 | 0.0018 | 15.1 | -3.9 | ubiquitin carboxyl-terminal hydrolase 14-like isoform 2 |
| CUST_243_PI406199769 | combined_c1228 | 0.0003 | 15.0 | -3.9 | NA |
| CUST_4211_PI406199772 | CD577261.1 | 0.0007 | 14.8 | -3.9 | NA |
| CUST_15806_PI406199769 | combined_c8386 | 0.0001 | 14.6 | -3.9 | NA |
| CUST_23965_PI406201128 | AGAP012280-RA | 0.0014 | 14.6 | -3.9 | isoform c |
| CUST_2037_PI406199769 | combined_c1027 | 0.0003 | 14.1 | -3.8 | AGAP003778-PA [Anopheles gambiae] |
| CUST_3420_PI406199769 | combined_c1728 | 0.0003 | 14.0 | -3.8 | NA |
| CUST_2036_PI406199769 | combined_c1027 | 0.0019 | 13.7 | -3.8 | AGAP003778-PA [Anopheles gambiae] |
| CUST_7527_PI406199769 | combined_c3809 | 0.0012 | 13.1 | -3.7 | juvenile hormone-inducible protein |
| CUST_321_PI406199769 | combined_c1629 | 0.0019 | 13.1 | -3.7 | hypothetical protein AND_21766 [*Anopheles darlingi*] |
| CUST_3711_PI406199769 | combined_c1873 | 0.0004 | 13.0 | -3.7 | monkey king protein |
| CUST_3054_PI406199772 | CD577873.1 | 0.0003 | 11.7 | -3.5 | pyruvate dehydrogenase e1 component subunit alpha |
| CUST_2615_PI406199772 | CD578093.1 | 0.0006 | 11.1 | -3.5 | AGAP003778-PA [*Anopheles gambiae*] |
| CUST_4583_PI406199772 | CD577072.1 | 0.0007 | 11.0 | -3.5 | atp synthase f0 subunit 6 |
| CUST_4534_PI406199772 | CD577097.1 | 0.0010 | 10.7 | -3.4 | atp synthase f0 subunit 6 |
| CUST_2616_PI406199772 | CD578093.1 | 0.0022 | 10.4 | -3.4 | AGAP003778-PA [*Anopheles gambiae*] |
| CUST_3377_PI406199772 | CD577703.1 | 0.0003 | 10.3 | -3.4 | AGAP005611-PA [*Anopheles gambiae*] |
| CUST_4214_PI406199772 | CD577260.1 | 0.0059 | 10.2 | -3.4 | cytochrome-b protein |
| CUST_4522_PI406199772 | CD577104.1 | 0.0010 | 10.2 | -3.3 | ATP synthase f0 subunit 6 |
| CUST_6673_PI406199798 | AGAP007593-RB | 0.0006 | 10.1 | -3.3 | glycerol-3-phosphate dehydrogenase |
| CUST_7526_PI406199769 | combined_c3809 | 0.0012 | 10.1 | -3.3 | juvenile hormone-inducible protein |
| CUST_21562_PI406201128 | AGAP010193-RA | 0.0021 | 10.0 | -3.3 | lectin 4 c-type lectin |
| CUST_5686_PI406199769 | combined_c2878 | 0.0008 | 10.0 | -3.3 | NADH dehydrogenase |
| CUST_8666_PI406199769 | combined_c4388 | 0.0068 | 9.9 | -3.3 | Serpin (srpn8) protein |
| CUST_2110_PI406199769 | combined_c1065 | 0.0010 | 9.7 | -3.3 | NA |
| CUST_8134_PI406199769 | combined_c4118 | 0.0004 | 9.7 | -3.3 | sodium-dependent excitatory amino acid transporter |
| CUST_9000_PI406199769 | combined_c4560 | 0.0004 | 9.7 | -3.3 | NA |

NA: Not available
