# Supplementary material for: A single mutation in the GSTe2 gene allows tracking of metabolically based insecticide resistance in a major malaria vector
Source: Genome Biol. 2014 Feb 25;15(2):R27. doi: 10.1186/gb-2014-15-2-r27 (PMC4054843; doi:10.1186/gb-2014-15-2-r27)
Supplement: Additional file 3: Table S2 — Genetic variability parameters for GSTe2 for resistant (alive) and susceptible (dead) mosquitoes fromKpome (Benin). [file gb-2014-15-2-r27-S3.doc]

**Table S2: Genetic variability parameters for alive and dead GSTe2 Kpome (Benin)**

| **Kpome** | **N** | **S** | **h** | **Syn** | **NSyn** | **π (k)** | **D** | **D*** |
| --- | --- | --- | --- | --- | --- | --- | --- | --- |
| **Alive** | 12 | 0 | 1 | 0 | 0 | 0.0 (0.0) | / | / |
| **dead** | 12 | 5 | 2 | 2 | 1 | 0.0025 (2.4) | 1.7^ns^ | 1.26^ns^ |
| **Total** | 24 | 5 | 2 | 2 | 1 | 0.0016 (1.44) | 0.23^ns^ | 1.16^ns^ |

N= number of sequences (2n); S, number of polymorphic sites; Syn, Synonymous mutations; Nsyn, Non-synonymous mutations; π, nucleotide diversity (k= mean number of nucleotide differences); D and D* Tajima’s and Fu and Li’s statistics; ns, not significant
