# Supplementary material for: A single mutation in the GSTe2 gene allows tracking of metabolically based insecticide resistance in a major malaria vector
Source: Genome Biol. 2014 Feb 25;15(2):R27. doi: 10.1186/gb-2014-15-2-r27 (PMC4054843; doi:10.1186/gb-2014-15-2-r27)
Supplement: Additional file 4 — A PDF document containing supplemental results and discussion. [file gb-2014-15-2-r27-S4.pdf]

Supplementary Results and Discussion

1-Genome-wide transcription microarray analysis

Up-regulated genes: Beside the main candidate genes mentioned in the main text, other genes were up-regulated in Benin including a mediator complex gene (FC34) which exhibited the highest fold-change. This gene encodes a multiprotein complex that functions as a transcriptional co-activator in all eukaryotes [1], but no known involvement in insecticide resistance has been previously reported. Another detoxification gene was an alpha-esterase ortholog of AGAP006700 (COEAE1G) in *An. gambiae* (FC5.1). This gene family is more involved in resistance to carbamates or organophosphates. Therefore, the up-regulation of this COEAE1G gene could be associated with the bendiocarb resistance observed in the Pahou mosquitoes [2]. However, this hypothesis requires confirmation through further functional analyses. Other probes belonging to genes with known association with insecticide resistance were also up-regulated in Pahou (Table S1). Among these genes were several probes that belong to cuticle protein genes, such as the adult-specific cuticular protein acp-20 (FC5.2) ortholog of AGAP006897 in *An. gambiae* (Table S1). Many probes belonging to different short-chain dehydrogenases were also up-regulated, notably the ortholog of AGAP005166 in *An. gambiae* (combined_c738; FC26). Other genes that were up-regulated include the ATP-binding cassette (ABC) transporter gene *ABCB7* (combined_c1762; FC2.8), an odorant receptor gene ortholog of AGAP001012 (FC8.2) and an odorant-binding protein (OBP4, FC2.7). Another up-regulated gene was glycoprotein 93 (FC11.6), whose ortholog in *Drosophila* is an ortholog of a mammalian heat shock protein gp96 [3], indicating that this gene could protect mosquitoes against the oxidative stress that could potentially result from exposure to insecticides.

The up-regulation of genes belonging to gene families such as ABC transporters, odorant receptors, cuticular proteins, heat shock proteins, serine proteases, short-chain dehydrogenases and UDP-glucosyltransferases is a common observation in genome-wide transcriptional profiling of insecticide resistant strains. Indeed, such up-regulation has previously been observed in different insect species such as *D. melanogaster* [4], *An. gambiae* [5, 6], *Ae. aegypti* [7] and *An. funestus* [8], with genes from these families involved in the various steps of the metabolism pathways of insecticides.

Down-regulated genes: The most down-regulated probe belonged to the cytochrome c oxidase subunit 3 (CD577188; FC44.3), and this down-regulation is supported by the fact that several probes belonging to this gene were also down-regulated. Other down-regulated probes include several probes for cytochrome b gene (CD577249; FC20.4) and a monkey king protein (combined_c1873; FC 18) (Table S2).

2-Genetic diversity of GSTe2 across Africa

Haplotype distribution of GSTe2 across Africa: A total of 46 *An. funestus* mosquitoes (2n=92) were analyzed across Africa and revealed 37 polymorphic sites, of which eight were singletons (Table 2). One insertion/deletion (T/-) was observed in intron 1. A total of 39 haplotypes were observed for the full gene (Figure S3B), 16 for coding regions (Figure S3C) and 7 protein variants (Figure S3D). When considering the full gene, the resistant haplotype BN23 was predominant, accounting for 34.8% of the total sample due to its nearly fixed frequency in Benin (95.8%) and its presence in Ghana (37.5%) and Cameroon (21.4%). The susceptible haplotype MAL3 was the next highest-frequency haplotype, with 14.1% mainly due to its predominance in

Malawi (44.4%) but also due to its broader geographical distribution, with a presence in Mozambique (20%), Cameroon (14.3%) and Uganda (10%). In addition, 31 of the 39 haplotypes were singletons, which is indicative of a high haplotypic diversity of GSTe2 across Africa (0.78>hd< 0.97) but not in Benin, where very low haplotype diversity was observed (hd=0.08). When considering only the coding region, the frequency of these two haplotypes increased particularly for the susceptible MAL3, which increased from 14.1 to 33.7% while the BN23 increased from 34.8 to 39.1% (Figure S3C). Their frequencies were even higher when only analyzing the non-synonymous substitutions, with 45.6% for the original BN23 and 43.7% for the MAL3 haplotype (Figure S3D).

3-Structural basis of DDT resistance conferred by GSTe2

Attempts to obtain the structure of another allele susceptible L119-GSTe2 from Malawi were not successful. The crystal structure of BN-GSTe2 allele was successfully determined without (apo-GSTe2) and with the co-factor glutathione (GSH) (holo-GSTe2), whereas for the UG allele, only the holo structure was determined.

Description of the GSH site: The GSH molecule lies in a polar cavity with its γ-glutamyl region forming hydrogen bonds with the Glu67 side chain, the main chain amide and the hydroxyl group of Ser68 and the positively charged side chain of the Arg112. The cysteinyl moiety forms two hydrogen bonds with the Ile55 carbonyl and amide main chain. Finally, the glycyl moiety establishes hydrogen bonds with the His53 side chain and main chain and with the Arg112 side chain. Several water molecules also stabilize GSH in the cavity. A 3D hydrogen-bond network stabilizes

the cofactor into an extended reactive conformation that enhances the formation of the thiolate anion ($-S^-$) in the active GS^- (Figure S6).

References

1. Taatjes DJ: **The human Mediator complex: a versatile, genome-wide regulator of transcription.** *Trends Biochem Sci* 2010, **35:**315-322.

2. Djouaka R, Irving H, Tukur Z, Wondji CS: **Exploring Mechanisms of Multiple Insecticide Resistance in a Population of the Malaria Vector Anopheles funestus in Benin.** *PLoS One* 2011, **6:**e27760.

3. Morales C, Wu S, Yang Y, Hao B, Li Z: **Drosophila glycoprotein 93 Is an ortholog of mammalian heat shock protein gp96 (grp94, HSP90b1, HSPC4) and retains disulfide bond-independent chaperone function for TLRs and integrins.** *J Immunol* 2009, **183:**5121-5128.

4. Pedra JH, McIntyre LM, Scharf ME, Pittendrigh BR: **Genome-wide transcription profile of field- and laboratory-selected dichlorodiphenyltrichloroethane (DDT)-resistant Drosophila.** *Proc Natl Acad Sci U S A* 2004, **101:**7034-7039.

5. Mitchell SN, Stevenson BJ, Muller P, Wilding CS, Egyir-Yawson A, Field SG, Hemingway J, Paine MJ, Ranson H, Donnelly MJ: **Identification and validation of a gene causing cross-resistance between insecticide classes in Anopheles gambiae from Ghana.** *Proc Natl Acad Sci U S A* 2012, **109:**6147-6152.

6. Vontas J, Blass C, Koutsos AC, David JP, Kafatos FC, Louis C, Hemingway J, Christophides GK, Ranson H: **Gene expression in insecticide resistant and susceptible Anopheles gambiae strains constitutively or after insecticide exposure.** *Insect Mol Biol* 2005, **14:**509-521.

7. Bariami V, Jones CM, Poupardin R, Vontas J, Ranson H: **Gene amplification, ABC transporters and cytochrome P450s: unraveling the molecular basis of pyrethroid resistance in the dengue vector, Aedes aegypti.** *PLoS Negl Trop Dis* 2012, **6:**e1692.

8. Riveron JM, Irving H, Ndula M, Barnes KG, Ibrahim SS, Paine MJ, Wondji CS: **Directionally selected cytochrome P450 alleles are driving the spread of**

pyrethroid resistance in the major malaria vector Anopheles funestus. *Proc Natl Acad Sci U S A* 2013, **110:**252-257.
