# Supplementary material for: A single mutation in the GSTe2 gene allows tracking of metabolically based insecticide resistance in a major malaria vector
Source: Genome Biol. 2014 Feb 25;15(2):R27. doi: 10.1186/gb-2014-15-2-r27 (PMC4054843; doi:10.1186/gb-2014-15-2-r27)
Supplement: Additional file 5: Figure S2 — Detection of the 119 F GSTe2 resistance allele in An. funestus. (A) The results of the TaqMan diagnostic assay for genotyping L119F, with three genotypes unambiguously identified (three clusters). (B) The genotype distribution of L119F alleles across nine countries in Africa shows there is a strong correlation between L119F genotypes and known patterns of DDT resistance. For example, the 119 F (T/T) genotype is fixed in the highly DDT-resistant Benin population but is completely absent in the fully susceptible southern African populations (Malawi, Mozambique and Zambia). (C) Assessment of the correlation between the L119F alleles and the permethrin (type I pyrethroid)-resistant phenotype. (D) Assessment of the correlation between the L119F alleles and the lambda-cyhalothrin (type II pyrethroid)-resistant phenotype. [file gb-2014-15-2-r27-S5.doc]

**Additional file 5: Figure S2.** Detection of the 119 F *GSTe2* resistance allele in An. funestus. (A) The results of the TaqMan diagnostic assay for genotyping L119F, with three genotypes unambiguously identified (three clusters). (B) The genotype distribution of L119F alleles across nine countries in Africa shows there is a strong correlation between L119F genotypes and known patterns of DDT resistance. For example, the 119 F (T/T) genotype is fixed in the highly DDT-resistant Benin population but is completely absent in the fully susceptible southern African populations (Malawi, Mozambique and Zambia). (C) Assessment of the correlation between the L119F alleles and the permethrin (type I pyrethroid)-resistant phenotype. (D) Assessment of the correlation between the L119F alleles and the lambda-cyhalothrin (type II pyrethroid)-resistant phenotype.
