# Supplementary material for: A single mutation in the GSTe2 gene allows tracking of metabolically based insecticide resistance in a major malaria vector
Source: Genome Biol. 2014 Feb 25;15(2):R27. doi: 10.1186/gb-2014-15-2-r27 (PMC4054843; doi:10.1186/gb-2014-15-2-r27)
Supplement: Additional file 6: Figure S3 — Metabolic activity of GSTe2. (A) DDT metabolism by GSTe2 from Benin has a high peak for the DDE metabolite product. (B) There is a reduction in the peak for permethrin metabolism by 119 F-GSTe2 in the reaction with GSTe2 (blue) compared to the control (black) (three replicates) using isocratic conditions. The arrow indicates potential metabolites. (C) Additional permethrin metabolism by the Benin GSTe2 enzyme using gradient conditions. Blue peaks refer to the metabolism profile of active GSTe2 whereas black peaks refer to the bovine serum incubation mixture (negative control). The arrows indicate the three potential metabolites. The analytes were eluted with a linear increase gradient program. [file gb-2014-15-2-r27-S6.doc]

**Additional file 6: Figure S3.** Metabolic activity of GSTe2. (A) DDT metabolism by GSTe2 from Benin has a high peak for the DDE metabolite product. (B) There is a reduction in the peak for permethrin metabolism by 119 F-GSTe2 in the reaction with GSTe2 (blue) compared to the control (black) (three replicates) using isocratic conditions. The arrow indicates potential metabolites. (C) Additional permethrin metabolism by the Benin GSTe2 enzyme using gradient conditions. Blue peaks refer to the metabolism profile of active GSTe2 whereas black peaks refer to the bovine serum incubation mixture (negative control). The arrows indicate the three potential metabolites. The analytes were eluted with a linear increase gradient program.
