# Supplementary material for: A single mutation in the GSTe2 gene allows tracking of metabolically based insecticide resistance in a major malaria vector
Source: Genome Biol. 2014 Feb 25;15(2):R27. doi: 10.1186/gb-2014-15-2-r27 (PMC4054843; doi:10.1186/gb-2014-15-2-r27)
Supplement: Additional file 7: Table S3 — Selection parameters for GSTe2 across Africa. [file gb-2014-15-2-r27-S7.doc]

**Table S3: Selection parameters of GSTe2 across Africa**

|  | **MK** | | | |  |  | **HKA** | | **dN/dS and Z test** | | | | **Ka/Ks** | | |
| --- | --- | --- | --- | --- | --- | --- | --- | --- | --- | --- | --- | --- | --- | --- | --- |
|  | **Fixed polymorphic between species** | | **Polymorphic within species** | | **NI** | **P value** | **X^2^** | **P value** | **dN** | **dS** | **dN/dS** | **P value** | **Ka x10^3^** | **Ks x10^3^** | **Ka/ks** |
|  | **Syn** | **NSyn** | **Syn** | **NSyn** |  |  |  |  |  |  |  |  |  |  |  |
| **Benin** | 92 | 24 | 0 | 1 | nc | 0.21 | 0.97 | 0.32 | 0.083 | 0.0 | Und | 0.14 | 0.017 | 0.0 | Und |
| **Cameroon** | 92 | 23 | 3 | 2 | 2.7 | 0.27 | 1.22 | 0.27 | 0.68 | 0.94 | 0.72 | 1.0 | 0.13 | 0.59 | 0.22 |
| **Ghana** | 91 | 23 | 3 | 1 | 1.2 | 1.0 | 0.0 | 0.98 | 0.46 | 0.88 | 0.52 | 1.0 | 0.091 | 0.55 | 0.16 |
| **Mozambique** | 92 | 23 | 2 | 0 | 0.0 | 1.0 | 0.0 | 0.99 | 0.0 | 0.4 | 0.0 | 1.0 | 0.0 | 0.25 | 0 |
| **Malawi** | 92 | 23 | 2 | 2 | 4 | 0.19 | 0.0 | 0.99 | 0.57 | 0.48 | 1.18 | 1.0 | 0.11 | 0.30 | 0.37 |
| **Uganda** | 92 | 23 | 0 | 3 | nc | 0.009 | 0.25 | 0.61 | 0.93 | 0.0 | Und | 0.059 | 0.18 | 0.0 | Und |
| **Total** | 90 | 23 | 7 | 6 | 3.3 | 0.074 | 0.04 | 0.84 | 2.2 | 1.45 | 1.52 | 1.0 | 0.15 | 0.48 | 0.31 |

NI: neutrality index; MK; Mcdonald and Kreitman test; HKA: Hudson, Kreitman and Aguade test; Und: Undetermined
