# Supplementary material for: A single mutation in the GSTe2 gene allows tracking of metabolically based insecticide resistance in a major malaria vector
Source: Genome Biol. 2014 Feb 25;15(2):R27. doi: 10.1186/gb-2014-15-2-r27 (PMC4054843; doi:10.1186/gb-2014-15-2-r27)
Supplement: Additional file 8: Figure S4 — Polymorphism patterns of GSTe2 in Africa. (A) Plot of genetic diversity parameters of GSTe2 across Africa that indicates there is strong directional selection of GSTe2 in Benin mosquitoes. hd, haplotype diversity; π, nucleotide diversity. (B) Haplotypes of GSTe2 (coding and non-coding) across six countries in Africa with contrasting DDT phenotypes. The polymorphic positions are indicated with numbers above the nucleotide, and the haplotypes are labeled from 1 to 39 with preceding initials from the country where the haplotype is predominant. An asterisk (*) shows that the haplotype was observed in other countries. N is the number of individuals who share the haplotype. (C) The same but only considering coding regions. (D) The same but only considering non-synonymous substitutions providing the different protein variants of GSTe2 across Africa. [file gb-2014-15-2-r27-S8.doc]

**Additional file 8: Figure S4.** Polymorphism patterns of *GSTe2* in Africa. (A) Plot of genetic diversity parameters of GSTe2 across Africa that indicates there is strong directional selection of *GSTe2* in Benin mosquitoes. hd, haplotype diversity; π, nucleotide diversity. (B) Haplotypes of GSTe2 (coding and non-coding) across six countries in Africa with contrasting DDT phenotypes. The polymorphic positions are indicated with numbers above the nucleotide, and the haplotypes are labeled from 1 to 39 with preceding initials from the country where the haplotype is predominant. An asterisk (*) shows that the haplotype was observed in other countries. *N* is the number of individuals who share the haplotype. (C) The same but only considering coding regions. (D) The same but only considering non-synonymous substitutions providing the different protein variants of GSTe2 across Africa.
