# Supplementary material for: A single mutation in the GSTe2 gene allows tracking of metabolically based insecticide resistance in a major malaria vector
Source: Genome Biol. 2014 Feb 25;15(2):R27. doi: 10.1186/gb-2014-15-2-r27 (PMC4054843; doi:10.1186/gb-2014-15-2-r27)
Supplement: Additional file 9: Figure S5 — Haplotype network for the full-length GSTe2 (coding and non-coding regions) across Africa. The frequency of each haplotype is reflected by the size of its polygon. Each node represents a segregating mutation (the polymorphic position is given above the branches). The polygons in grey are resistant haplotypes that harbor the resistant 119 F allele whereas the haplotypes in white are susceptible. The mutational position of 499 is circled to indicate that this polymorphic position, which corresponds to L119F, is key to shaping the GSTe2 haplotype distribution. [file gb-2014-15-2-r27-S9.doc]

**Additional file 9: Figure S5.** Haplotype network for the full-length *GSTe2* (coding and non-coding regions) across Africa**.** The frequency of each haplotype is reflected by the size of its polygon. Each node represents a segregating mutation (the polymorphic position is given above the branches). The polygons in grey are resistant haplotypes that harbor the resistant 119 F allele whereas the haplotypes in white are susceptible. The mutational position of 499 is circled to indicate that this polymorphic position, which corresponds to L119F, is key to shaping the *GSTe2* haplotype distribution.
