# Supplementary material for: A single mutation in the GSTe2 gene allows tracking of metabolically based insecticide resistance in a major malaria vector
Source: Genome Biol. 2014 Feb 25;15(2):R27. doi: 10.1186/gb-2014-15-2-r27 (PMC4054843; doi:10.1186/gb-2014-15-2-r27)
Supplement: Additional file 10: Table S4 — Genetic differentiation using Kst. [file gb-2014-15-2-r27-S10.doc]

**Table S4: Genetic differentiation using *K_ST_***

|  | Malawi | Mozambique | Uganda | Benin | Cameroon |
| --- | --- | --- | --- | --- | --- |
| Malawi |  |  |  |  |  |
| Mozambique | 0.016^ns^ |  |  |  |  |
| Uganda | 0.082** | 0.113* |  |  |  |
| Benin | 0.492*** | 0.582*** | 0.497*** |  |  |
| Cameroon | 0.125*** | 0.126*** | 0.217*** | 0.340*** |  |
| Ghana | 0.130*** | 0.132*** | 0.167*** | 0.116*** | 0.047* |

PERMTEST calculates Hudson’s K_ST_ statistic of genetic differentiation. K_ST_ is equal to 12KS/KT, where KS is a weighted mean of K1 and K2 (mean number of differences between sequences in subpopulations 1 and 2, respectively) and KT represents the mean number of differences between two sequences regardless of their subpopulation. The null hypothesis of no genetic differentiation will be rejected (P<0.05) when KS is small and K_ST_ is close to 1.PM test; Probability obtained by the permutation test with 1000 replicates); ns, not significant; *, 0.01<P<0.05; **, 0.001<P<0.01; ***, P<0.001
