# Supplementary material for: A single mutation in the GSTe2 gene allows tracking of metabolically based insecticide resistance in a major malaria vector
Source: Genome Biol. 2014 Feb 25;15(2):R27. doi: 10.1186/gb-2014-15-2-r27 (PMC4054843; doi:10.1186/gb-2014-15-2-r27)
Supplement: Additional file 11: Figure S6 — The conformations of the GSH binding pocket (G-site) of the resistant BN-GSTe2 allele (purple) and the susceptible UG-GSTe2 allele (green) have no significant differences. The GSH cofactor and the protein amino acids and water molecules hydrogen-bonded to it are represented in ball-and-stick mode. [file gb-2014-15-2-r27-S11.doc]

**Additional file 11: Figure S6.** The conformations of the GSH binding pocket (G-site) of the resistant BN-GSTe2 allele (purple) and the susceptible UG-GSTe2 allele (green) have no significant differences**.** The GSH cofactor and the protein amino acids and water molecules hydrogen-bonded to it are represented in ball-and-stick mode.
