# Supplementary material for: A single mutation in the GSTe2 gene allows tracking of metabolically based insecticide resistance in a major malaria vector
Source: Genome Biol. 2014 Feb 25;15(2):R27. doi: 10.1186/gb-2014-15-2-r27 (PMC4054843; doi:10.1186/gb-2014-15-2-r27)
Supplement: Additional file 12: Figure S7 — Comparative analysis of the H-site structure of different GSTe2 alleles and agGSTd1-6. The size and shape of the site are key determinants of DDT metabolism capacity. (Top) The molecular surface representations of those residues constituting the H-site of Uganda-GSTe2 (green), Benin-GSTe2 (purple), agGSTe2 (white) and agGSTd1-6 (yellow). Oxygen and nitrogen atoms at the surface are depicted in red and blue, respectively. (Bottom) Overlays of the structures of Uganda-GSTe2 (green) with Benin-GSTe2 (purple), agGSTe2 (white) and agGSTd1-6 (yellow) showing that most differences are in the secondary structural elements related to the H-site. [file gb-2014-15-2-r27-S12.doc]

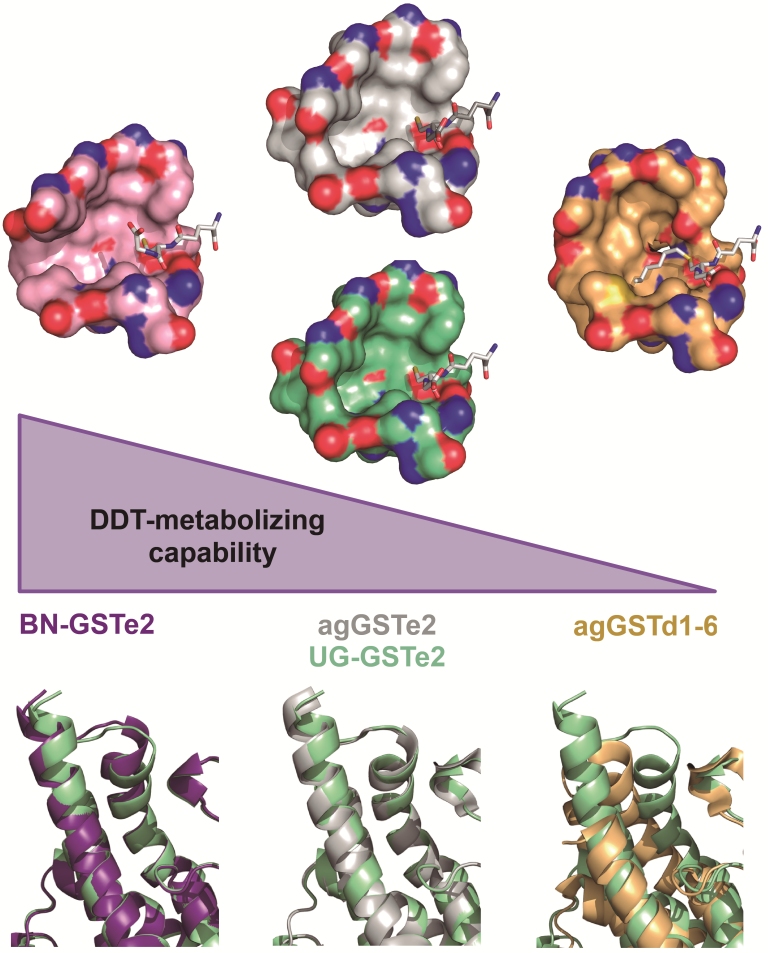


**Additional file 12: Figure S7.** Comparative analysis of the H-site structure of different GSTe2 alleles and agGSTd1-6. The size and shape of the site are key determinants of DDT metabolism capacity. (Top) The molecular surface representations of those residues constituting the H-site of Uganda-GSTe2 (green), Benin-GSTe2 (purple), agGSTe2 (white) and agGSTd1-6 (yellow). Oxygen and nitrogen atoms at the surface are depicted in red and blue, respectively. (Bottom) Overlays of the structures of Uganda-GSTe2 (green) with Benin-GSTe2 (purple), agGSTe2 (white) and agGSTd1-6 (yellow) showing that most differences are in the secondary structural elements related to the H-site.
