# Supplementary material for: A single mutation in the GSTe2 gene allows tracking of metabolically based insecticide resistance in a major malaria vector
Source: Genome Biol. 2014 Feb 25;15(2):R27. doi: 10.1186/gb-2014-15-2-r27 (PMC4054843; doi:10.1186/gb-2014-15-2-r27)
Supplement: Additional file 13: Table S5 — List of primers used in this study. [file gb-2014-15-2-r27-S13.doc]

**Table S5: List of primers used in this study**

|  | **Forward primer** | **Reverse primer** | | **Expected size (bp)** |
| --- | --- | --- | --- | --- |
| **qRT-PCR primers** | | | | |
| **GSTe2** | GTTTGAAGCAGTTGCCATACTACGAGG | | TCAAGCTTTAGCATTTTCCTCCTTTTTGGC | 101 |
| **CYP6P9a** | CAGCGCGTACACCAGATTGTGTAA | | TCA CAA TTT TTC CAC CTT CAA GTA ATT ACC CGC | 92 |
| **CYP6P9b** | CAGCGCGTACACCAGATTGTGTAA | | TTA CAC CTT TTC TAC CTT CAA GTA ATT ACC CGC | 97 |
| **Combined_c738** | GCAGGTGACCCATAGTCGTT | | GCTGTTGAGGGAATGTTACGA | 136 |
| **RSP7** | GTGTTCGGTTCCAAGGTGAT | | TCCGAGTTCATTTCCAGCTC | 98 |
| **Actin** | TTAAACCCAAAAGCCAATCG | | ACCGGATGCATACAGTGACA | 111 |
| **Primers for functional analysis** | | | | |
| **GSTe2** | AGATCTATGACCAAGCTAGTTCTGTACACGCT | | TCTAGATCAAGCTTTAGCATTTTCCTCCTTTTTGGC |  |
|  | GAATTCCATATGACCAAGCTAGTTCTGTACACGCT | | CCGCTCGAGTCAAGCTTTAGCATTTTCCTCCTTTTTGGC |  |
| **Primers used for amplification of GSTe2 for polymorphism analysis** | | | | |
| **GSTe2Full** | ATGACCAAGCTAGTTCTGTACACGCT | | TCAAGCTTTAGCATTTTCCTCCTTTTTGGC | 666bp for cDNA; 881bp with introns |

In red is restriction site for *Bgl*II; in green is for *Xba*I; blue is for *Nde*I; purple is for *Xho*I
