# Supplementary material for: A single mutation in the GSTe2 gene allows tracking of metabolically based insecticide resistance in a major malaria vector
Source: Genome Biol. 2014 Feb 25;15(2):R27. doi: 10.1186/gb-2014-15-2-r27 (PMC4054843; doi:10.1186/gb-2014-15-2-r27)
Supplement: Additional file 14: Table S6 — Primers for TaqMan L119F GSTe2 assay. [file gb-2014-15-2-r27-S14.doc]

**Table S6: Primers for TaqMan L119F GSTe2 assay**

| **Primers** | **Sequence** | **Modification** |
| --- | --- | --- |
| **Forward** | AACAATTTTTCATTTCTTATTCTCATTTAC |  |
| **Reverse** | CGACTCGATCTTCGGGAATGTC |  |
| **reporter L119** | AGGAGCGTATTCTTTTCTA | VIC |
| **Reporter 119F** | AGGAGCGTATTTTTTTCTA | FAM |
