# Supplementary material for: A single mutation in the GSTe2 gene allows tracking of metabolically based insecticide resistance in a major malaria vector
Source: Genome Biol. 2014 Feb 25;15(2):R27. doi: 10.1186/gb-2014-15-2-r27 (PMC4054843; doi:10.1186/gb-2014-15-2-r27)
Supplement: Additional file 15: Table S7 — Summary statistics for data collection, processing and model refinement. [file gb-2014-15-2-r27-S15.doc]

**Table S7: Summary statistics of data collection, processing and model refinement**

| **Crystal data** | **holo_ BN-GSTe2** | **holo_UG-GSTe2** |
| --- | --- | --- |
| Space group | *P* 2_1_ | *P* 2_1_2_1_2_1_ |
| Cell dimensions |  |  |
| *a*, *b*, *c* (Å) | 47.15, 99.62, 89.90 | 49.25, 73.04, 129.65 |
| α, β, γ (°) | 90.0, 99.2, 90.0 | 90.0, 90.0, 90.0 |
| *Z* | 4 | 2 |
| V_M_ (Å3 Da-1) | 2.00 | 2.33 |
| Solvent content (%) | 38.66 | 47.29 |
| Diffraction protocol |  |  |
| Radiation source | ESRF (ID 14.4) | ESRF (ID 23.2) |
| Wavelength (Å) | 0.94 | 0.87 |
| Detector type | ADSC Q4 CCD | MARCCD |
| X ray beam size (microns) | 100 | 10 |
| Crystal to detector distance | 258.25 | 189.0 |
| Dp increment per image | 1.0 | 1.0 |
| Temperature (K) | 100 | 100 |
| Data collection statistics |  |  |
| Resolution (Å) | 46.55-2.20 (2.32-2.20) | 32.48-1.64 (1.64-1.73) |
| No of unique reflections | 38035 | 58184 |
| No of observed reflections | 254696 | 691132 |
| Completeness (%) | 91.6 (85.1) | 99.7 (99.4) |
| Multiplicity | 6.7 (6.6) | 6.1 (5.8) |
| Mean I / σ(I) | 11.5 (5.0) | 9.0 (3.4) |
| R_sym_, R_pim_ | 0.146 (0.555), 0.059 (0.224) | 0.13 (0.70), 0.054 (0.26) |
| Refinement |  |  |
| Resolution (Å) | 46.55-2.20 | 32.48-1.64 |
| R_work_ / R_free_ | 0.18 / 0.22 | 0.19 / 0.21 |
| No. atoms | 7064 | 4100 |
| Number of reflections | 97156 | 57924 |
| Model |  |  |
| Molecules per a.u. | 4 | 2 |
| Aminoacids | 842 | 437 |
| Water molecules | 275 | 577 |
| Cofactor molecules (GSH) | 2 | 2 |
| Average B-factors | 27.0 | 15.0 |
| R.m.sdeviations |  |  |
| Bond lengths (Å) | 0.009 | 0.021 |
| Bond angles (°) | 1.21 | 1.94 |
| Ramachandran Plot statistics | 97.6 % in the core  1.8 % in the allowed  0.6 % outliers | 98.4 % in the core  1.1 % in the allowed  0.5 % outliers |
